# Spodium Bonds in Biological Systems: Expanding the Role of Zn in Protein Structure and Function

**DOI:** 10.1021/acs.jcim.1c00594

**Published:** 2021-08-10

**Authors:** Himansu S. Biswal, Akshay Kumar Sahu, Antonio Frontera, Antonio Bauzá

**Affiliations:** † School of Chemical Sciences, National Institute of Science Education and Research (NISER), Bhimpur-Padanpur, Via—Jatni, Khurda, 752050 Bhubaneswar, India; ‡ Training School Complex, Homi Bhabha National Institute, Anushakti Nagar, 400094 Mumbai, India; § Department of Chemistry, Universitat de les Illes Balears, Crta. de Valldemossa km 7.5, 07122 Palma (Baleares), Spain

## Abstract

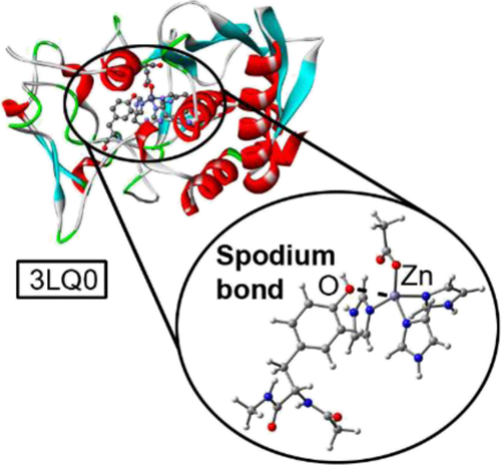

Understanding the structural and functional implications of metal ions is of pivotal significance to chemical biology. Herein, we report first time the evidence of spodium bonds (SpB’s, an attractive noncovalent force involving elements from group 12 and electron-rich species) in tetrahedral Zn-binding sites. Through a combined crystallographic (PDB analysis) and computational (*ab initio* calculations) study, we demonstrate that Zn SpB’s are abundant and might be involved in protein structure and enzyme inhibition.

## Introduction

During the last decade, noncovalent interactions (NCIs) have starred a fast growing revolution which have led them to become essential resources of the chemist toolbox owing to their crucial role in several fields of modern chemistry, such as supramolecular chemistry,^1^ molecular recognition,^2^ and materials science.^3^ Despite of the great importance that hydrogen-bonding (HB) interactions play in many chemical and biological systems,^4,5^ such as in enzymatic chemistry and protein folding and binding phenomena,^6^ other NCIs based on the p-block of elements [aerogen (Ae),^7^ halogen (Hal),^8^ chalcogen (Ch),^9^ pnictogen (Pn),^10^ and tetrel (Tr) bonds]^11^ have emerged during the last decade. This family of interactions is also known as “σ-hole bonding”. They are based on electropositive potential regions located on the extension of covalent X–Ae, X–Hal, X–Ch, X–Pn, and X–Tr bonds, which are able to interact in a favorable manner with electron-rich species (i.e., a lone pair, an anion, or a π-system). Their study and recognition by the scientific community have led to powerful and novel applications in the fields of rational drug design,^12−14^ molecular aggregation,^15−17^ or even tuning self-assembly phenomena,^18−20^ among others.

**Figure 1:**
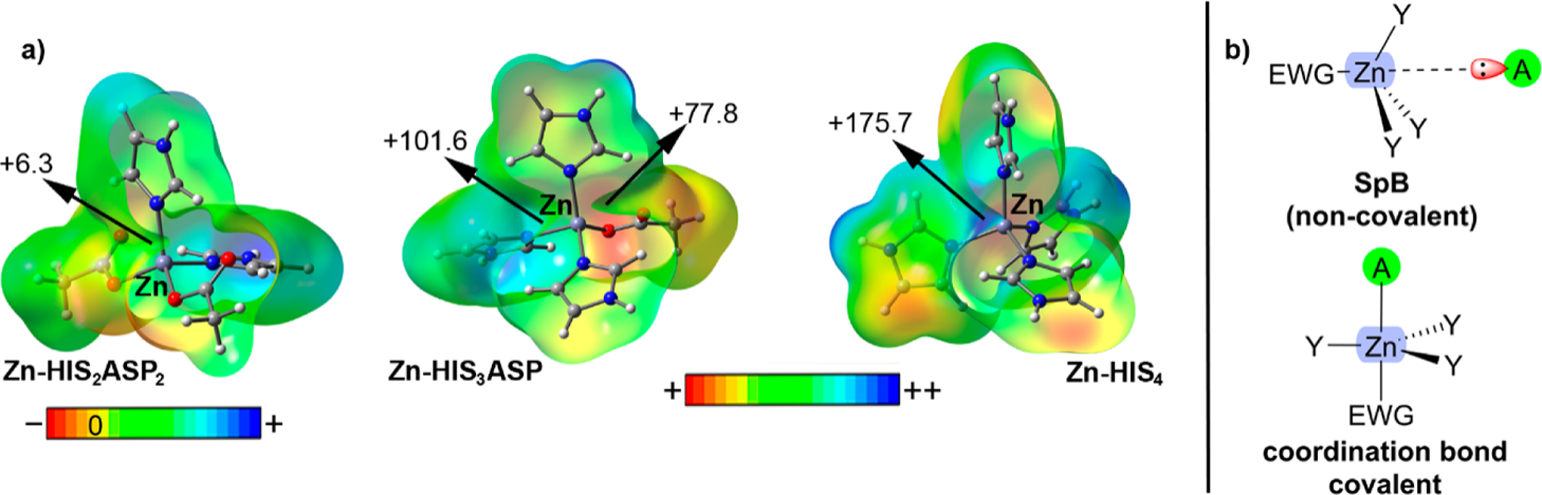
(a) MEP surfaces of three different Td Zn sites with a net charge of 0 (Zn–HIS_2_ASP_2_), +1 (Zn–HIS_2_ASP), and +2 (Zn–HIS_4_), HIS were modeled as imidazole rings and ASP as acetate. Energy values at concrete regions of the surface are indicated in kcal·mol^–1^ (0.002 au). (b) Schematic representation of a SpB vs a common coordination bond. EWG stands for the electron-withdrawing group.

In biology, Zn^2+^ is one of the most important trace metal ions and an essential cofactor in many metabolic enzymes and regulatory proteins.^21−23^ Zn^2+^ can play a catalytic role acting as a Lewis acid (i.e., an electron pair acceptor), thus facilitating deprotonation of Zn-coordinated water in human carbonic anhydrase II (HCA2)^24^ or stabilizing negatively charged intermediate species, such as in carboxypeptidase A.^25^ On the other hand, Zn^2+^ ions also play a fundamental role in protein structure and folding, such as in Zn-finger proteins.^26^ Very recently, some of us have demonstrated that group 12 elements (Zn, Cd, and Hg), when are in their common +2 oxidation state and (pseudo)tetrahedral coordination environment, present a region of positive electrostatic potential (known as σ-hole^27^) suitable to interact with a lone pair-bearing partner, thus coining the term spodium bond (SpB). This can be further understood by means of a molecular electrostatic potential (MEP) analysis shown in Figure 1a. As noted, three MEP surfaces corresponding to Zn–HIS_2_ASP_2_ (Zn(0)), Zn–HIS_3_ASP (Zn(I)), and (Zn–HIS_4_) (Zn(II)) are shown. Zn N–HIS ligands were modeled as imidazole rings while O-ASP and O-GLU ligands were modeled as acetate. Interestingly, in all cases, positive MEP values over the Zn atom (located along the Zn–N or Zn–O bond axis) were observed, which are prototypical descriptors of a σ-hole. The Zn MEP values show a dramatic increase ongoing from a total charge of 0 (+6.3 kcal·mol^–1^) to +1 (+101.6 and +77.8 kcal·mol^–1^) and finally to +2 (+175 kcal mol^–1^). These results are useful to understand (i) the strong influence of the Zn-coordinated moieties on its σ-hole donor ability and (ii) the importance of the spatial ligand disposition around the metal center, since it is crucial for the accessibility of Zn’s σ-hole.

In this context, the nature of SpB’s is markedly different from coordination and (pseudo)coordination bonds, both in strength and directionality (see Figure 1b).^28^ Although this interaction is on its mere naissance, it has achieved a rapid recognition among the scientific community, owing to the number of theoretical and experimental works published to date.^29−34^ Herein, we report first-time evidence of the existence of Zn SpB’s in biological systems through an inspection of the PDB combined with theoretical calculations (RI-MP2/def2-TZVP level of theory). Concretely, we carried out a PDB survey to find tetrahedral Zn^2+^ centers involved in Sp-bonding interactions. From these, 13 structures exhibiting highly directional SpB’s were selected for QM calculations to unravel new insights on the strength of the interaction, using AIM and NCIplot analyses to further characterize the interaction. Finally, the biological implications of two selected examples were discussed in order to highlight the importance of the interaction in (i) the catalytic mechanism of Zn-dependent proteins and (ii) the stabilization of Zn-tetrahedral binding sites.

## Methods

### PDB Analysis

Only structures containing Zn with a resolution below 2 Å in the Protein Data Bank^35^ (PDB) were considered during the search (October 2020 release), resulting in 6911 PDB files. The following structural parameters were considered to classify the Zn···A (A = N, O, and S) contact as a SpB

- distance between Zn and A: 2.5 Å ≤ *d*_Zn···A_ < 5 Å
- angle: 140° ≤ θ(∠Y–Zn···A) ≤ 180° where Y, A = (N, O, and S).

We searched four Y atoms bonded with Zn to maintain tetrahedral geometry. To avoid the possibility of Zn–Y coordination bonds to be considered as covalent bonds, we restricted the distance between Zn and Y to less than 2.5 Å for the search, which is greater than their sum of covalent radii.^36^ The covalent radii considered for Zn, N, O, and S were 1.22, 0.71, 0.66, and 1.05 Å, respectively. On the other hand, the Bondi van der Waals radii^37^ of Zn, N, O, and S were taken as 1.39, 1.55, 1.52, and 1.8 Å, respectively. It is important to note that Bondi’s van der Waals radii for Zn is short (1.39 Å) and similar to its covalent radius (1.22 Å). Thus, zinc’s van der Waals radii seem largely underestimated, as also suggested by several investigations.^38−41^ Consequently, using different Zn vdW radii (e.g., 2.39 Å as proposed by Alvarez and collaborators)^42^ would result in most Zn···A contacts falling within the sum of the van der Waals radii. To process large pools of data within a reasonable timeframe, we used an in-home program written in Python designed to execute batch processing of the PDB files. Finally, Stride program^43^ was used to assign secondary structures to the amino acid residues involved in spodium bonding.

### Modeling and Calculation of SpB’s in PDB Structures

#### Phase-1: Selection of PDBs for Theoretical Calculations

During the modeling phase, only those structures exhibiting highly directional SpB’s (∠Y–Zn···A comprised between 160 and 180°) were analyzed. After manual inspection of each structure, 13 PDBs were selected for calculations. Finally, if several SpB’s were present in the same X-ray structure, only the one exhibiting the shortest Zn···A (A = O/S) distance was selected for calculations.

#### Phase-2: Creating the Computational Models

**Figure 2:**
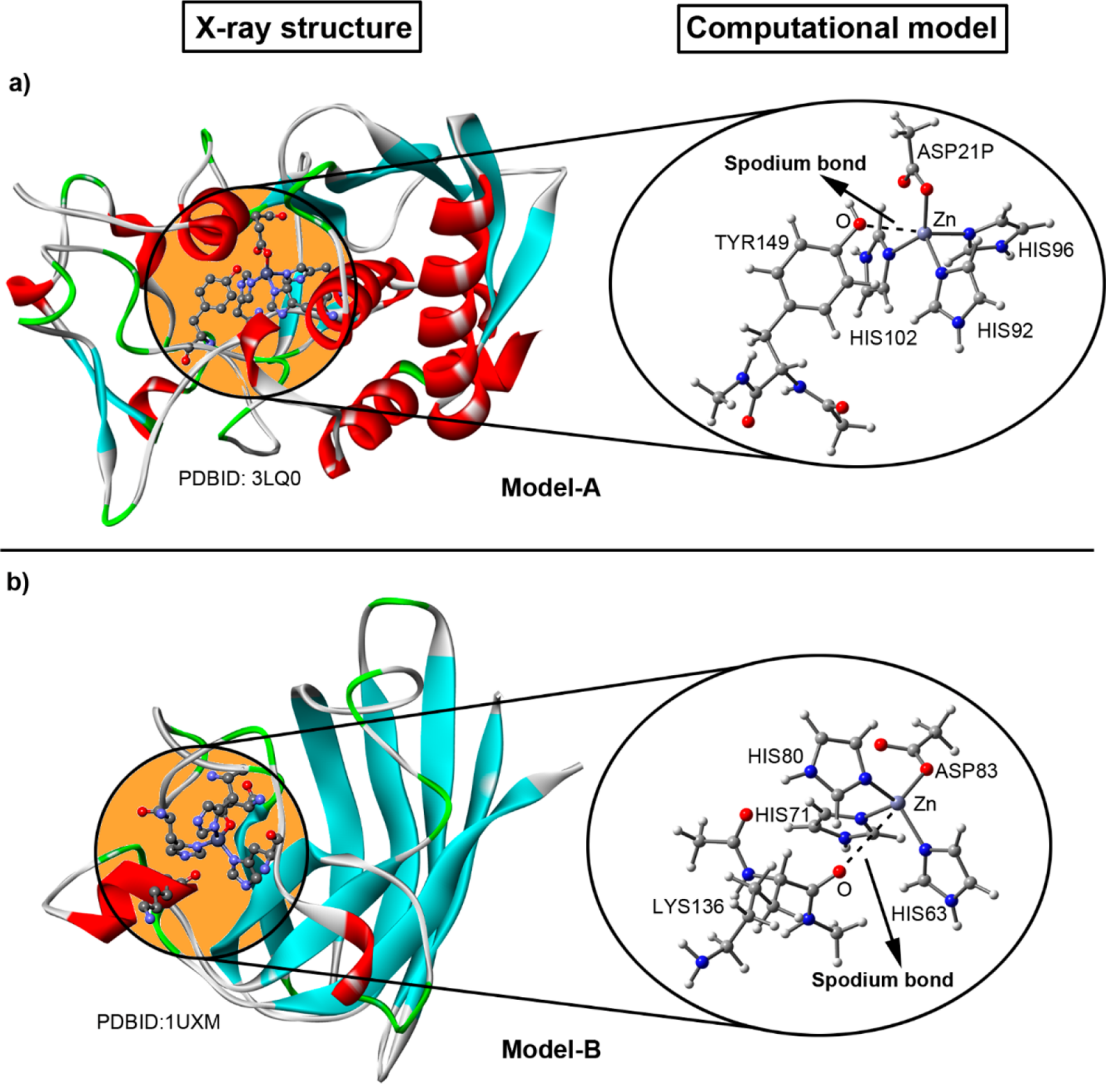
Reference computational models A and B used for the calculation of SpB’s. Partial views of the X-ray crystal structures of 3LQ0 (a) and 1UXM (b) exhibiting SpB’s (left) and theoretical models used (right); these include the Zn and its tetrahedral coordination environment and the interacting electron-rich partner [an amino acid residue (model A) or an O atom belonging to the carbonyl group of the protein backbone (model B)]. The SpB’s are represented by the dashed lines connecting the O atoms and the Zn center. The PDBIDs are also included.

**Figure 3:**
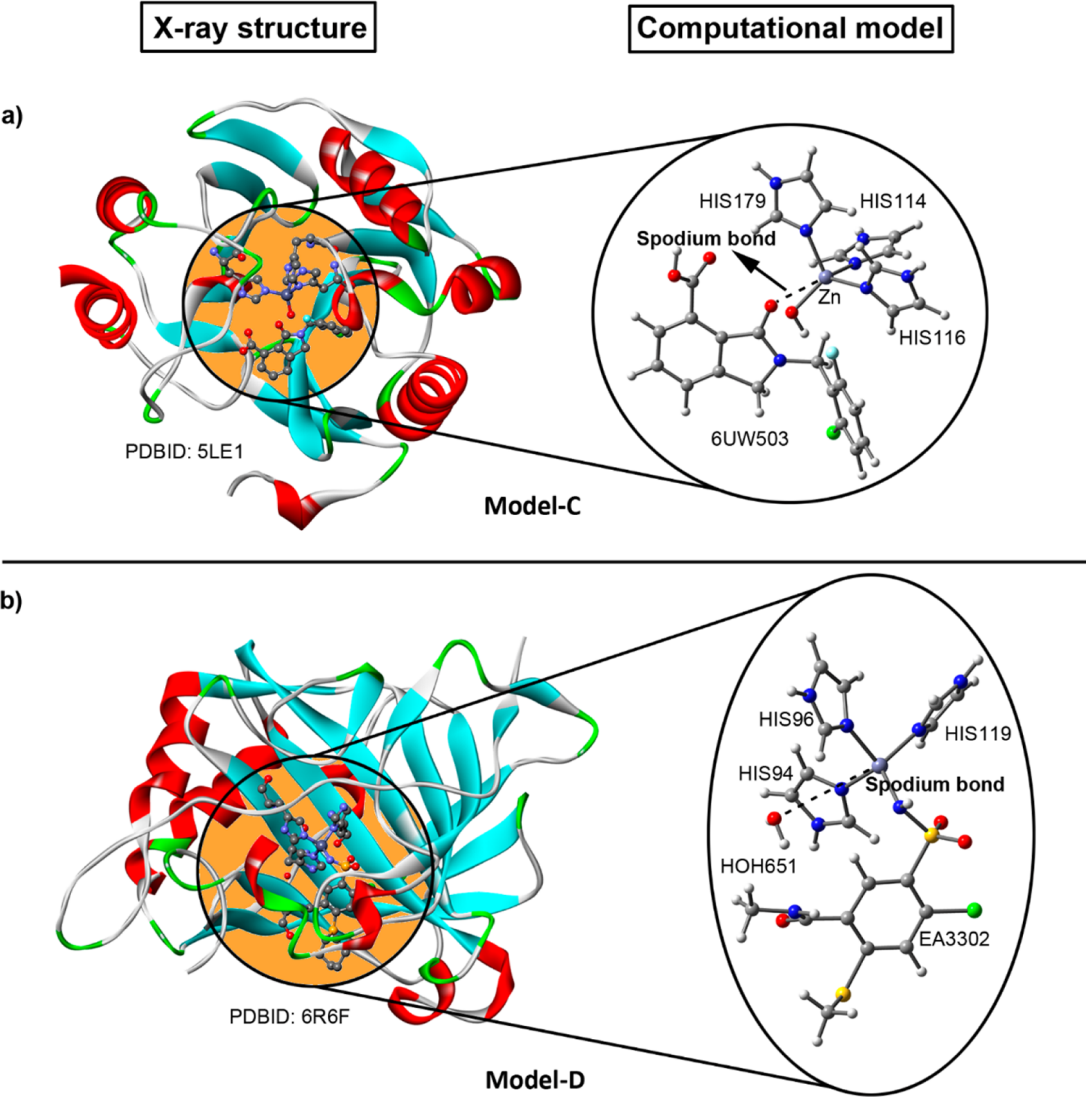
Reference computational models C and D used for the calculation of SpB’s. Partial views of the X-ray crystal structures of 5LE1 (a) and 6R6F (b) exhibiting SpB’s (left) and theoretical models used (right); these include the Zn atom and its coordinated residues and the interacting electron-rich species [an O atom belonging to an artificial ligand (model C) or a water molecule (model D)]. The SpB’s are represented by the dashed lines connecting the O atoms and the Zn center. The PDBIDs are also included.

A theoretical model of the Zn metal center and the interacting partner for each PDB structure was manually elaborated. Depending on the interacting moiety (e.g., an amino acid residue, an artificial ligand, or a water molecule), we can distinguish between four building schemes, as indicated in Figures 2 and 3 (see also Tables 1–4 for more details).

**Table 1:** Interacting Amino Acid (AA) and Zn-Coordinated Environment (Coordres) Included in the Computational Models of 3LQ0, 4P4F, 4G9L, 4MDG, 2CBA, 6QNG, 3MHC, 5JN8, 6FE0, and 2O6E Structures (Model A)

PDB ID	AA	Coordres
3LQ0	TYR	3HIS and 1ASP
4P4F^a^	GLU	1HIS, 2ASP and 1 phosphorus amide
2O6E^b^	MET	3HIS and 1ASP

a An O atom from the phosphorus amide was protonated to ensure neutrality of the Zn center.

b The protein chain from the MET residue was replaced by a methyl group to properly evaluate the spodium-bonding interaction.

**Table 2:** Interacting AA and Zn-Coordinated Environment (Coordres) Included in the Computational Models of 2ZNR, 3F1A, 1UXM, 1F18, and 2VR7 Structures (Model B)

PDB ID	AA	Coordres
2ZNR^a^	LYS	3HIS and 1CYS
3F1A^a^	HIS	3HIS and 1ASP
1UXM	LYS	3HIS and 1ASP
1F18^a^	LYS	3HIS and 1CYS
2VR7^b^	LYS	3HIS and 1CYS

a The lateral chain of the AA was replaced by a methyl group to properly evaluate the spodium-bonding interaction.

b The residue group from the LYS residue was replaced by a methyl group to avoid evaluating ancillary HB interactions.

**Table 3:** Interacting Species (LigandID) and Zn-Coordinated Environment (Coordres) Included in the Computational Models of 5LE1 and 6SJ4 Structures (Model C)

PDB ID	ligand ID	Coordres
5LE1^a^	6UW503	3HIS and a OH group
6SJ4^b^	LFK501	3HIS and 1ASP

a Only one Zn(II) center was considered and the N–Zn–N–C dihedral angle involving HIS114 and HIS179 imidazole rings rotated 90° to properly evaluate the SpB. The carboxylate moiety of the inhibitor was considered as protonated.

b The carboxylate moiety of the inhibitor was considered as protonated.

**Table 4:** Interacting Water Molecule (WaterID) and Zn-Coordinated Environment (Coordres) Included in the Computational Models of 5UUD, 5A0X, and 6R6F Structures (Model D)

PDB ID	water ID	Coordres
5UUD	HOH520	2HIS, 1ASP, and 1 water molecule
5A0X	HOH2120	2HIS, 1ASP, and 1PRO
6R6F^a^	HOH651	3HIS and 1 sulfonamide group

a The N atom from the sulfonamide group coordinated to Zn was considered as deprotonated.

Bearing these data in mind, we manually took the Zn and its coordinating residues and the interacting partner (AA, water, or ligand) using the following procedure:

Zn coordinated ligands were modeled as follows:

- HIS residues as imidazole rings.
- ASP/GLU residues as acetate groups.
- CYS residues as thiomethyl groups.

**Figure 4:**
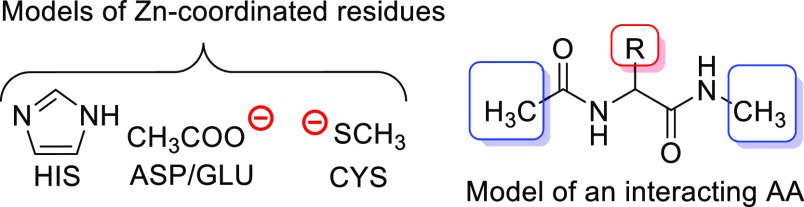
Computational models of the Zn-coordinated residues and the interacting amino acid.

Electron-rich donors were modeled by capping the amino and carboxyl ends of the interacting AA with methyl groups (see Figure 4).

The theoretical models were created using Accelrys Discovery Studio Visualizer^44^ and Gaussian-16 calculation package.^45^ Once created, all H atoms were optimized at the BP86^46^-D3^47^/def2-SVP^48^ level of theory, to obtain a more reliable position before evaluating the interaction energy of the system, and the rest of the atoms were kept frozen during the optimization process. After proper H relaxation, the SpB interaction energies were calculated by means of single-point calculations at the RI-MP2^49^/def2-TZVP^48^ level of theory (using TURBOMOLE 7.0 program).^50^ The optimization of the whole system leads to a different structure where other forces (mainly HB interactions) and coordination bonds are predominant, thus being not useful for evaluating the SpB interaction energies. The modifications performed on some structures to properly evaluate the Sp-bonding interactions highlight the complexity of choosing a general computational protocol that fits into a proper balance between reliability and accuracy, due to the intricate combination of NCIs that take part during the Zn(II) protein complexation process.

#### Phase-3: Calculating SpB Interaction Energies

The following computational techniques are in line with the state-of-the-art theoretical methods available in the computational chemist toolbox. Particularly, the binding energies of the spodium complexes were calculated following the supermolecule approximation, that is, as the energy difference between the complex and the isolated monomers (Δ*E*_complex_ = *E*_complex_ – *E*_Zn center_ – E_electron-rich partner_), at the RI-MP2/def2-TZVP level of theory by means of the program TURBOMOLE. Owing to the absence of benchmarking studies due to the novelty of the interaction, the RI-MP2 method combined with the TZVP basis set was chosen since it achieved success to accurately represent other σ-hole-based interaction energies involving both neutral and charged electron donors.^51^ To obtain the values of *E*_Zn center_ and *E*_electron-rich partner_, their corresponding geometries were subtracted from the complex and their energies were evaluated separately at the RI-MP2/def2-TZVP level of theory. The interaction energies were calculated with correction for the basis set superposition error (BSSE) using the Boys–Bernardi counterpoise technique.^52^ The MEP surfaces have been computed at the RI-MP2/def2-TZVP level of theory and the wave function analyses at the B3LYP^46,53−55^-D3/def2TZVP level of theory using the Gaussian-16 calculation package.

The topological properties of electron density were analyzed using the QTAIM methodology. A brief description of some relevant concepts within Bader’s topology analysis is appropriate to facilitate the analysis of the results. The existence of a bond path connecting two nuclei implies that the two atoms are bonded to one another. Such a path is characterized by the bond critical point (BCP), which is the point exhibiting a minimum charge density along the bond, but a maximum along the directions perpendicular to the bond path. A critical point can be characterized by the number of zero eigenvalues of the associated Hessian matrix and the algebraic sum of their signs, which determine its rank and its signature, respectively. A BCP is denoted as (3, −1) and has one positive (λ_3_) and two negative (λ_1_ and λ_2_) curvatures, one (λ_3_) associated with the charge density along the bond path and the other two (λ_1_ and λ_2_) perpendicular to the bond path. There can be other types of nondegenerate critical points: (3, −3), (3, +1), and (3, +3). The first corresponds to the position of local maxima of the charge density (the nuclei). The two other types occur as a consequence of particular geometrical arrangements of bond paths and define elements of the molecular structure. If the bond paths are linked so as to form a ring of bonded atoms, a (3, +1) ring critical bond is formed in the interior of the ring. If the bond paths are arranged as to enclose the interior of a molecule with ring surfaces, then a (3, +3) cage critical point is found in the interior of the cage, the charge density being a local minimum at such a point. The characteristics of the BCP were discussed in terms of the electron density (ρ) and its Laplacian (∇^2^ρ). Finally, the NCIplot^56^ index allows convenient visualization of both inter- and intramolecular interactions in real space. It plots isosurfaces of the reduced-density gradient (RDG, related to |∇|/ρ^4/3^), which are colored in agreement to values of the electron density. The NCI contacts are characterized by the regions of small RDG at low densities, being mapped in real space by plotting an isosurface of *s* for a low value of RDG. Besides, the sign of the second eigenvalue of the density Hessian times the density is color-mapped onto the isosurfaces, which allows the characterization of both the strength and (un)favorable nature of the interactions. More precisely, the color scheme is composed by a red-yellow-green-blue scale using red for repulsive (ρ_cut_^+^) and blue for attractive (ρ_cut_^–^) NCI interaction density. Weak repulsive and weak attractive interactions are identified by yellow and green surfaces, respectively.

## Results and Discussion

### PDB Survey

**Figure 5:**
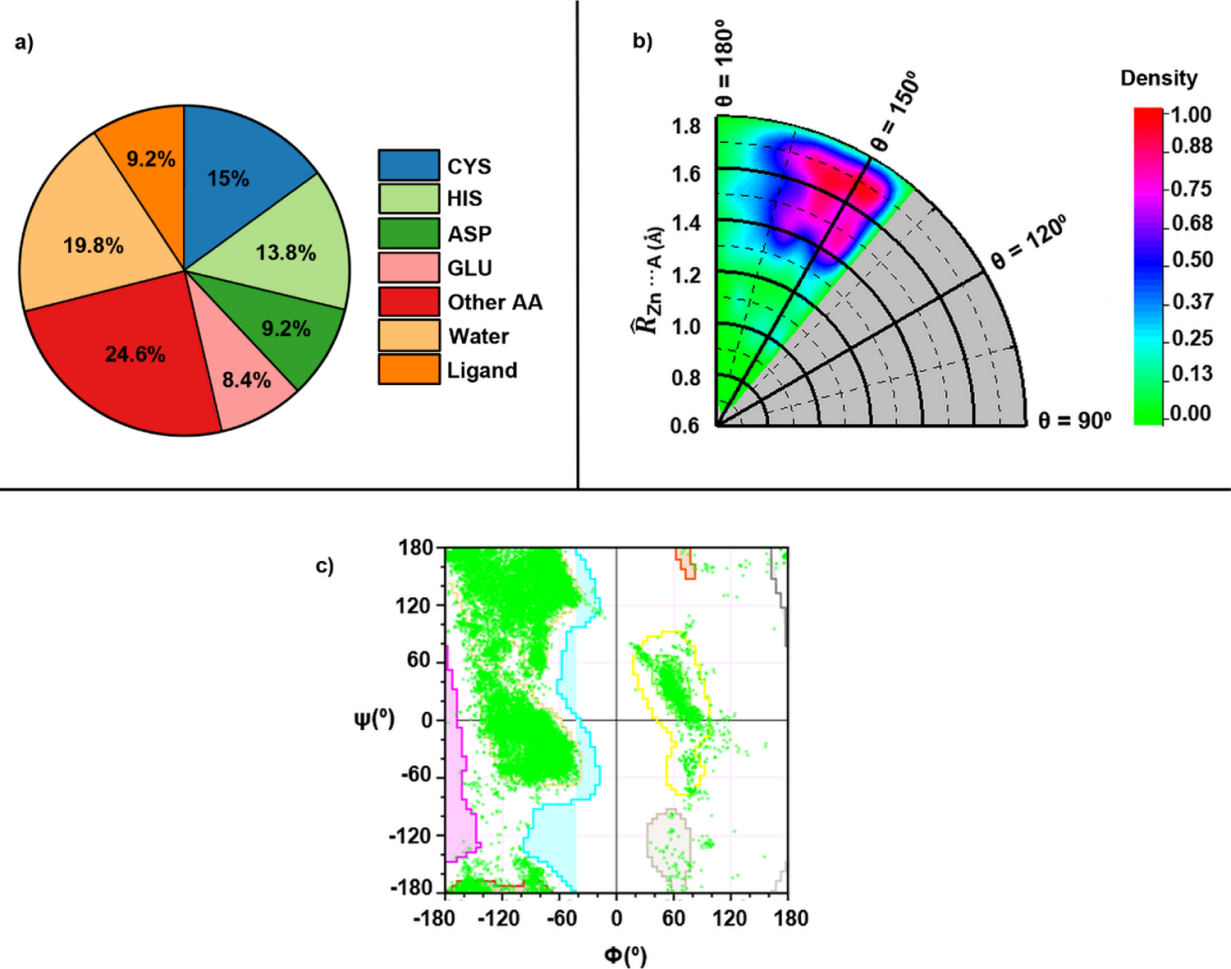
(a) Pie chart for electron-rich partners involved in the SpB. (b) Radial distribution of Zn···A SpB’s in proteins. The angle (θ) made by A with respect to the Zn–R bond is plotted against the distance of Zn to Zn to A (*R̂*_Zn···A_) atom, in which the sum of van der Waals radii of Zn and A atoms has been taken as the normalization factor. (c) Ramachandran plot of combined N/O/S atoms belonging to the amino acid residue.

In Figure 5, the results from an in-depth exploration of the PDB for noncovalent Td Zn(II)···A interactions (A = N, O, and S, see Supporting Information for detailed information regarding search criteria and statistical data) are also shown, revealing a total number of 52,758 contacts. From them, approximately 50% of Zn···A contacts can be attributed to CYS, HIS, ASP, and GLU residues, followed by other amino acids (∼25%), water molecules (∼20%), and nonprotein ligands (∼10%), as indicated in Figure 5a. Hence, SpB’s are a vastly extended interaction in Zn-dependent proteins and negatively charged residues (mainly CYS, ASP, and GLU) are those mostly involved as electron-rich partners. A more detailed picture analysis is shown in Table 5, particularly, in the case of nitrogen, most contacts involve the protein backbone; however, due to electron-pair delocalization, these are expected to be very weak SpB’s. On the other hand, the opposite is observed in the case of oxygen, which exhibits a predominance of contacts involving the amino acid side chain (SC). In the case of sulphur, the amino acid SC is obviously involved in all contacts. Furthermore, in the case of ligands, Zn···O interactions are the most abundant, followed by Zn···S and Zn···N SpB’s. Finally, Zn···O(water) and Zn···O(SC) SpB’s are equally abundant, which highlights the importance of water buried inside Zn active sites.

**Table 5:** Classification of Zn···N/O/S Contacts

	amino acid				
donor	backbone	side chain	ligand	water	total
N	13,779	7210	847		21,836
O	4870	10,994	3374	10,442	29,680
S		624	618		1242

Another interesting analysis is the radial distribution graphic shown in Figure 5b, which evidences that most of the Zn···A contacts are established at around 0.4 and 0.8 Å beyond the sum of the Zn···A vdW radii. Actually, this finding strongly supports the van der Waals radius for Zn(II) proposed by Alvarez,^22^ which is 1 Å longer (2.39 Å) than Bondi’s value (1.39 Å). Additionally, this plot also confirms that Zn SpB’s exhibit certain directionality since most of the SpB’s are established between 140 and 170°, in line with the results obtained from our previous CSD study.^28^ Finally, from the Ramachandran plot shown in Figure 5c, it can be deduced that a variety of secondary protein structures are involved in the formation of SpB’s. In particular, right-handed α-helices, collagen triple helix, and β-sheets are the most-abundant structural patterns, as indicated by the clusters located at the top-left and middle-left regions of the plot. Another interesting conclusion that can be extracted is the presence of left-handed α-helices (middle-right cluster), although being less abundant than the rest of structural motifs.

### Theoretical Study

To gain further knowledge on the strength and directionality of Zn SpB’s, a set of 13 PDB structures were selected for theoretical calculations (see Table 6 for more details). We were mainly interested in analyzing those structures exhibiting highly directional SpB’s (∠Y–Zn···A comprised between 160 and 180°) because they represent the most favorable situation in terms of the interaction between the Td Zn site and the electron-rich partner. In addition, only those SpB’s involving neutral electron donors were considered for calculations, in order to get rid of the strong electrostatic component that dominates the interaction when both counterparts (Zn center and electron donor molecule) are charged.

**Table 6:** List of PDB Codes (PDB ID), Interacting Residues (ResID), and Electron-Rich Atom (El.R.)^a^

PDB ID	ResID	Δ*E*	ΔE_BSSE_	*d*_Zn···_A	∠Y–Zn···A
2ZNR	LYS404	–11.6	–9.8	4.462	173.7
3F1A	HIS172	–12.8	–11.4	4.206	172.9
5LE1	6UW503	–16.2	–13.3	3.806	177.3
3LQ0	TYR149	–11.1	–8.5	3.011	175.9
5UUD	HOH520	–26.7	–24.1	3.193	177.2
5A0X	HOH2120	–17.6	–15.5	4.037	178.0
6SJ4	LFK501	–10.8	–6.4	3.628	172.3
4P4F	GLU425	–18.6	–14.5	3.506	173.2
1UXM	LYS136	–21.5	–19.0	3.777	179.2
1F18	LYS136	–11.4	–9.7	4.080	178.0
2VR7	LYS136	–15.6	–13.7	4.036	175.5
6R6F	HOH651	–9.5	–6.7	4.246	162.6
2O6E	MET117	–13.0	–10.6	3.128	175.2

a In addition, the uncorrected (Δ*E*, in kcal·mol^–1^) and BSSE-corrected interaction energy values (Δ*E*_BSSE_ in kcal·mol^–1^) and Zn···A distances (*d*_Zn···A_, in Å) and Y–Zn···A angles (in °) are also indicated. A is, in all cases, an O atom except for 2O6E where it is a S atom.

Each structure gathered in Table 6 shows a particular Zn coordination environment, although the most common ligands are HIS, ASP, and GLU residues, water molecules, and other nonprotein ligands (i.e., artificial molecules). The global charge of the Td Zn center is +1 in all complexes (except for 4P4F structure, which is 0), while the electron-rich molecules are neutral (e.g., MET and TYR residues or water). A close look to the results reveals some interesting points worthy to mention. First, the interaction energy values are attractive in all cases, ranging from moderate (−8.5 kcal·mol^–1^) to strong (−24.1 kcal·mol^–1^) values. Second, SpB’s are usually accompanied by ancillary noncovalent forces (e.g., H bonds or CH−π interactions) established between the electron-donor moiety and the Zn-coordinated residues that further contribute to binding. For instance, 5UUD, where a water molecule is acting as electron-donor moiety, obtained the most favorable binding energy value, also due to the establishment of two strong hydrogen bonds involving a Zn-coordinated water molecule and an acetate ligand. Also, in 1F18, a H bond is established between a CH group from a Zn-coordinated HIS residue and the O atom from the carbonyl group of LYS136. These ancillary interactions, although present, might function as a “molecular anchorage” for the electron-rich partner, prior to their interaction with the Td Zn center.

### AIM and NCIplot Analyses

**Figure 6:**
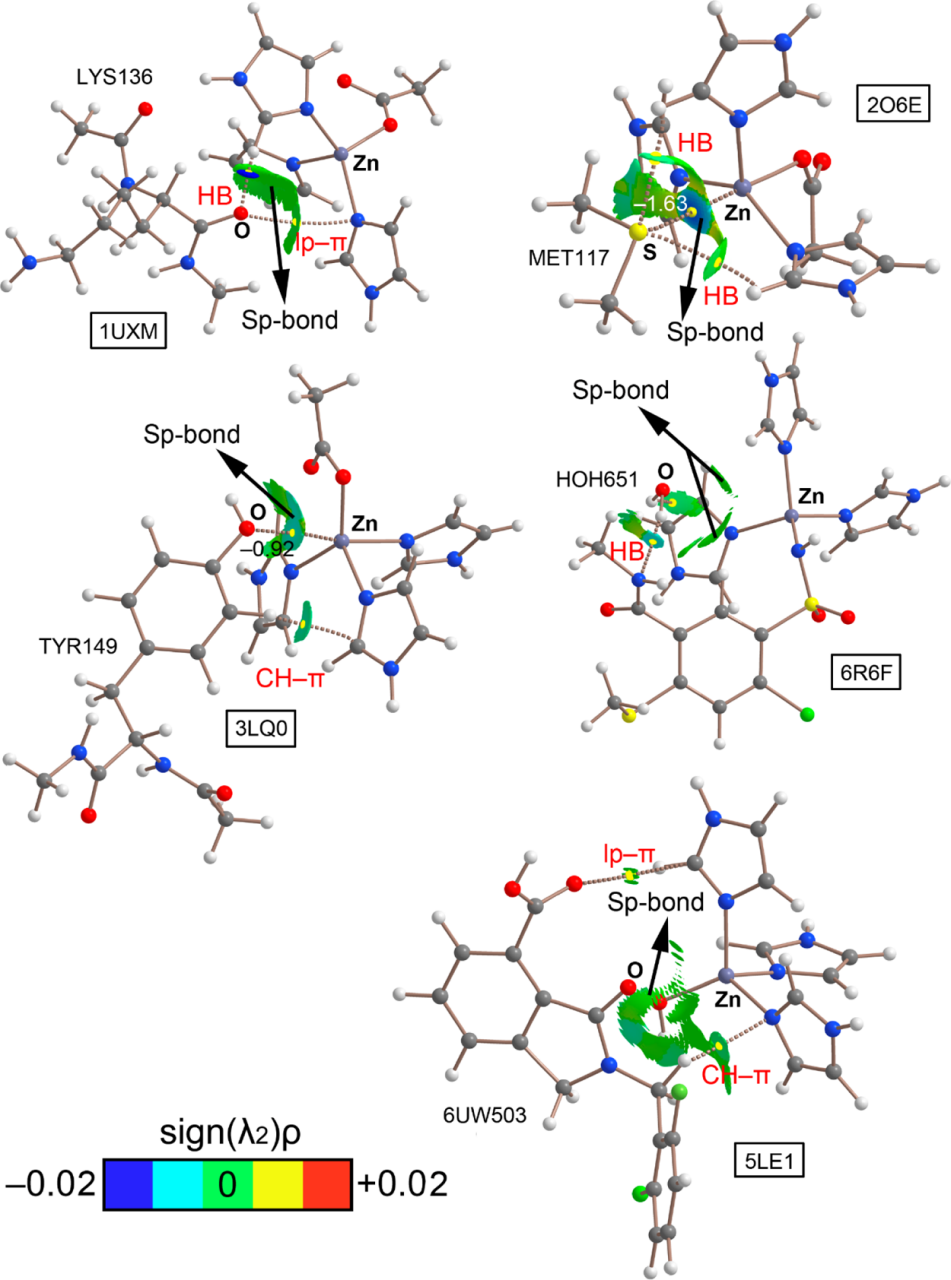
Distribution of intermolecular BCPs (yellow dots) and bond paths in the models of 1UXM (a), 2O6E (b), 3LQ0 (c), 6R6F (d), and 5LE1 (e) structures. Ancillary interactions are highlighted in red. The values of the density (ρ·10^2^) related to the Sp-bond interaction in 2O6E and 3LQ0 structures are also included in au NCIPlot color range −0.02 au ≤ (sign λ_2_)ρ ≤ +0.02 au.

To gain further insights on the SpB’s exhibited in this set of complexes, five representative examples involving O and S atoms as electron donors were taken for AIM and NCIplot analyses and the results are shown in Figure 6.

First, in the case of the 1UXM structure, the AIM analysis did not show a BCP connecting the O and Zn atoms. Instead, two BCPs connect the O atom from the carbonyl moiety of LYS136 to (i) an imidazole ring and (ii) a CH group from another imidazole moiety. However, the NCIplot analysis showed the presence of a green isosurface covering the space between the O and the Zn atoms, thus denoting the presence of an SpB. Second, in the 2O6E structure, a BCP connects the S atom with the Zn center, thus characterizing the SpB. In addition, two BCPs connect the S atom from MET117 to two CH groups belonging to Zn-coordinated imidazole rings. The presence of the SpB was further confirmed by the NCIplot analysis, showing a bluish isosurface between the S and Zn counterparts.

In the case of 3LQ0, two BCPs connect TYR149 with the Zn center. First, a BCP connects the O atom from the phenol ring to the Zn center, thus characterizing an SpB. Also, the NCIplot analysis reveals the presence of a green isosurface between the O and the Zn atoms which confirms the existence of the interaction. Second, a BCP connects a CH group from the phenol ring to the π-system of HIS92, thus characterizing an ancillary CH−π interaction. We have also included in Figure 6 the values of the ρ·10^2^ at the bond CPs that characterize the SpB’s for both 2O6E and 3LQ0 structures. As noted, the value of the density is larger in the former Sp-bond complex, which denotes a stronger interaction, in agreement with the results gathered from the energetic study and their corresponding NCIplot surface colors, thus giving reliability to the utilization of the NCIplot index as a qualitative method to characterize and visualize interactions. In the 6R6F structure, only two BCPs connecting the H atoms from the water molecule with vicinal imidazole rings are shown, thus characterizing two HB interactions. However, two greenish isosurfaces corresponding to the location of the O lone pairs are located between the O and the Zn atoms, thus denoting the presence of a weak Sp-bond interaction.

Finally, in the case of the 5LE1 structure, no BCP connecting the O atom (belonging to the carbonyl moiety of the ligand) with the Zn center was found; however, the NCIplot analysis reveals the presence of a green isosurface between both atoms, which evidences the existence of an SpB. In addition, two BCPs connect (i) an O atom (belonging to the carboxylate moiety) and (ii) a CH group from the inhibitor structure to the π-systems of HIS179 and HIS116, respectively, thus characterizing ancillary lone pair−π (Lp−π) and CH−π interactions.

### Biological Implications of SpB’s

**Figure 7:**
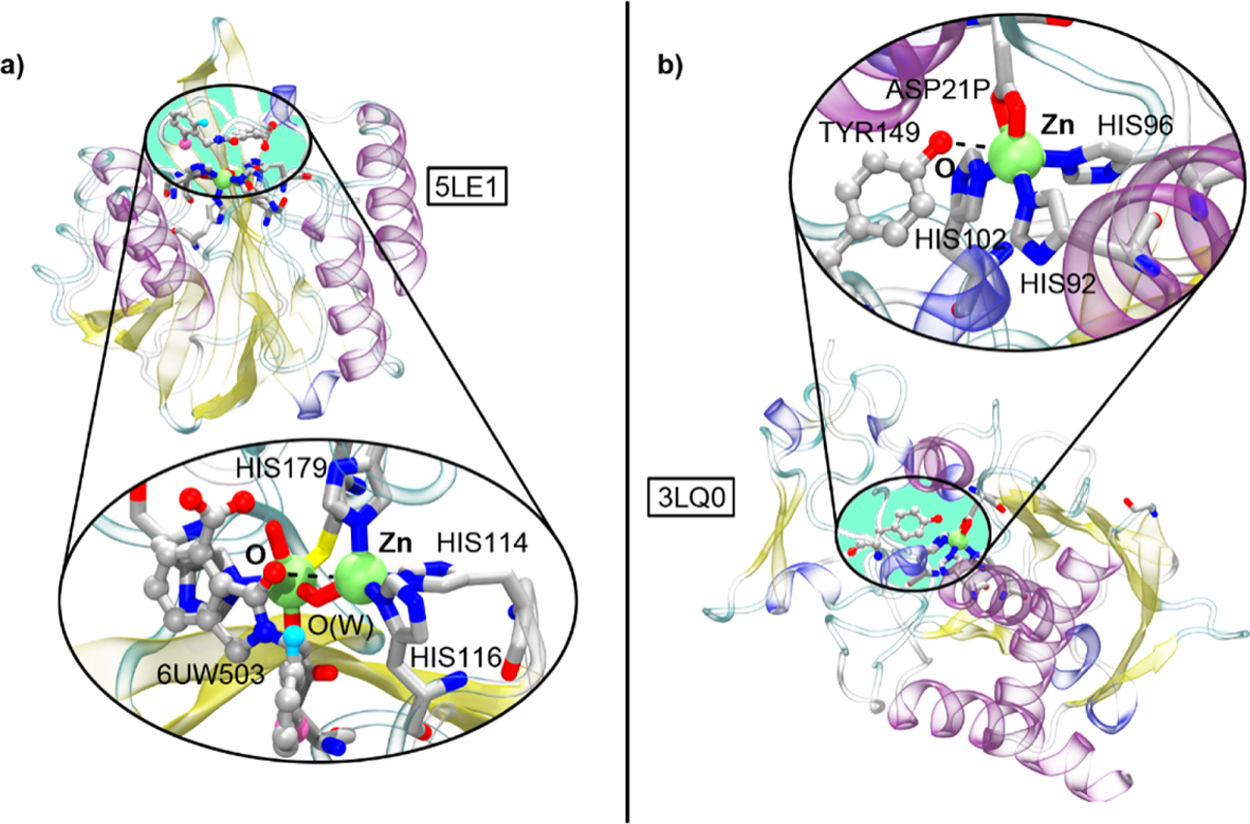
Partial views of 5LE1 (a) and 3LQ0 (b) X-ray structures. The Sp-bond interaction is magnified inside the circular parts of the figure.

To demonstrate the impact of this interaction in biological systems, two examples were selected where the concept of SpB’s can be evoked to understand enzyme inhibition and protein structure. The first example involves metallo-β-lactamases (MLBs), a family of enzymes involved in the mechanisms of resistance to β-lactam antibacterials by carrying out their hydrolysis, thus being considered as a potential therapeutic target.^57^ In a recent report,^58^ the Verona Integron-encoded (VIM-2) protein, which is a clinically important B1 class MBL, was used to develop a new family of MBL inhibitors. VIM-2 utilizes di-Zn(II), with both Zn ions having crucial roles in catalysis, with respect to β-lactam substrate binding and hydrolytic water activation.^59^ One of the compounds displaying the most-potent VIM-2 inhibition (IC_50_ value of 10.6 μM) showed noncovalent binding to Zn, as revealed by ^1^H CPMG (Carr–Purcell–Meiboom–Gill) NMR analyses. A close look to the diZn(II) metal center in the 5LE1 structure (Figure 7a) reveals the presence of a positive electrostatic potential region along the Zn–N_HIS114_ bond (+71.5 kcal·mol^–1^, see Figure S4a in Supporting Information), which facilitates the formation of an SpB between the O atom from the amide moiety of the inhibitor and Zn2 atom (*d* = 3.806 Å), with an N_HIS114_–Zn2–O_A_ angle of 177.3°. The calculated strength of the interaction resulted in −13.3 kcal mol^–1^, which represents around a 15% of a Zn–O bond energy value (70–80 kcal mol^–1^).^60^ This energetic value includes long-range interactions between the carboxylate moiety of the inhibitor and HIS179, which also contribute to binding. Hence, SpB’s are key players in the inhibition mechanism of VIM-2 and useful to understand the low IC_50_ value exhibited by the inhibitor in the 5LE1 structure. This example nicely illustrates the potential of this novel interaction for the development of new therapeutic agents targeting specific Zn-dependent proteins.

A second interesting example encompasses the structure and activation mechanism of metallopeptidases (MPs).^61^ This family of proteins carries out the cleavage of peptide bonds, which is an essential process for life,^62^ and so, their deregulation leads to several diseases ranging from cancer^63^ to neurological insults and cardiovascular disorders.^64^ In this vastly extended protein family, astacins and serralysins are two subtypes of MPs that present a catalytic site formed by the Zn coordination of 3HIS, one ASP, and a TYR residue, which is considered important from a structural and functional perspective (Figure 7b). More precisely, the TYR149 residue is implicated in a back and forth flipping movement during substrate anchoring, cleavage, and product release, which has been demonstrated in studies involving astacin-inhibitor or substrate-mimic structures,^65^ known as “tyrosine switch”. In 3LQ0 (free enzyme structure), TYR149 is establishing an SpB with the Zn catalytic center (Zn···O_TYR149_ distance of 3.010 Å and N_HIS96_–Zn–O_TYR149_ angle of 176°), which is facilitated by the presence of a region of positive electrostatic potential along the Zn–HIS96 axis (+89.1 kcal mol^–1^, see Figure S4b in Supporting Information). This SpB confers TYR149 mobility to flip back and forth upon substrate coordination (owing to its noncovalent nature) and it is replaced by a hydrogen bond that stabilizes the tetrahedral intermediate during the reaction cycle. Hence, SpB’s might be an important source of structural Zn stabilization before starting the catalytic cycle and noticeable contributors to understanding the flexibility of the “tyrosine switch” rearrangement during the enzyme’s reaction cycle. The calculated strength of the interaction resulted in −8.5 kcal mol^–1^, which is of similar magnitude than strong hydrogen bonds.

## Conclusions

SpB’s are an abundant noncovalent force in nature and generally unnoticed contributors to Zn–protein structure and functionality. Two selected examples showcased their importance in the inhibition of β-lactam antibacterials and in the catalytic cycle of metallopeptidases. Furthermore, a computational ab initio study on selected structures revealed qualitative information regarding the strength of the interaction and the influence of the Td Zn center charge on the stability of the complexes. With new Zn-dependent enzymes being discovered every year, the question whether SpB’s might become a pivotal binding force in biological systems seems tantalizing. We hope that the results gathered in this study will serve as a retrospective guide to expand the knowledge of Zn as a Lewis acid in chemical biology and to unveil the potential of SpB’s to chemical biology rational drug design fields.

## Supporting Information Available

Additional data regarding PDB statistical results and cartesian coordinates of the PDB structures used to compute the Sp-bond interactions studied herein (PDF)

## Notes

Protein Data Bank (https://www.rcsb.org/), Turbomole software (https://www.turbomole.org/), Gaussian 16 calculation package (https://gaussian.com/gaussian16/), AIMall (http://aim.tkgristmill.com/), and Stride program (http://webclu.bio.wzw.tum.de/stride/). All data to reproduce theoretical calculations are included in Supporting Information.
