# Supplementary material for: Spodium Bonds in Biological Systems: Expanding the Role of Zn in Protein Structure and Function
Source: J Chem Inf Model. 2021 Aug 10;61(8):3945–54. doi: 10.1021/acs.jcim.1c00594 (PMC8525862; doi:10.1021/acs.jcim.1c00594)
Supplement: Supplementary file 1 — ci1c00594_si_001.pdf [file ci1c00594_si_001.pdf]

Supporting Information

Spodium Bonds in Biological Systems: Expanding the Role of Zn in Protein Structure and Function.

Himansu Biswal*,[a,b] Akshay Kumar Sahu,[a,b] Antonio Frontera,[c] and Antonio Bauzá*,[c]

[a]School of Chemical Sciences, National Institute of Science Education and Research (NISER), PO- Bhimpur-Padanpur, Via-Jatni, District- Khurda, PIN - 752050, Bhubaneswar, India
[b]Homi Bhabha National Institute, Training School Complex, Anushakti Nagar, Mumbai 400094, India
E-mail: himansu@niser.ac.in*
[c]Department of Chemistry, Universitat de les Illes Balears, Crta. de Valldemossa km 7.5, 07122 Palma (Baleares), Spain; E-mail: antonio.bauza@uib.es*

Electronic Supplementary Information

Table of contents:

Statistical analyses of the PDB Page 2
Figure S4 Page 5
Cartesian Coordinates of the PDB models Page 6

Statistical analyses of the PDB

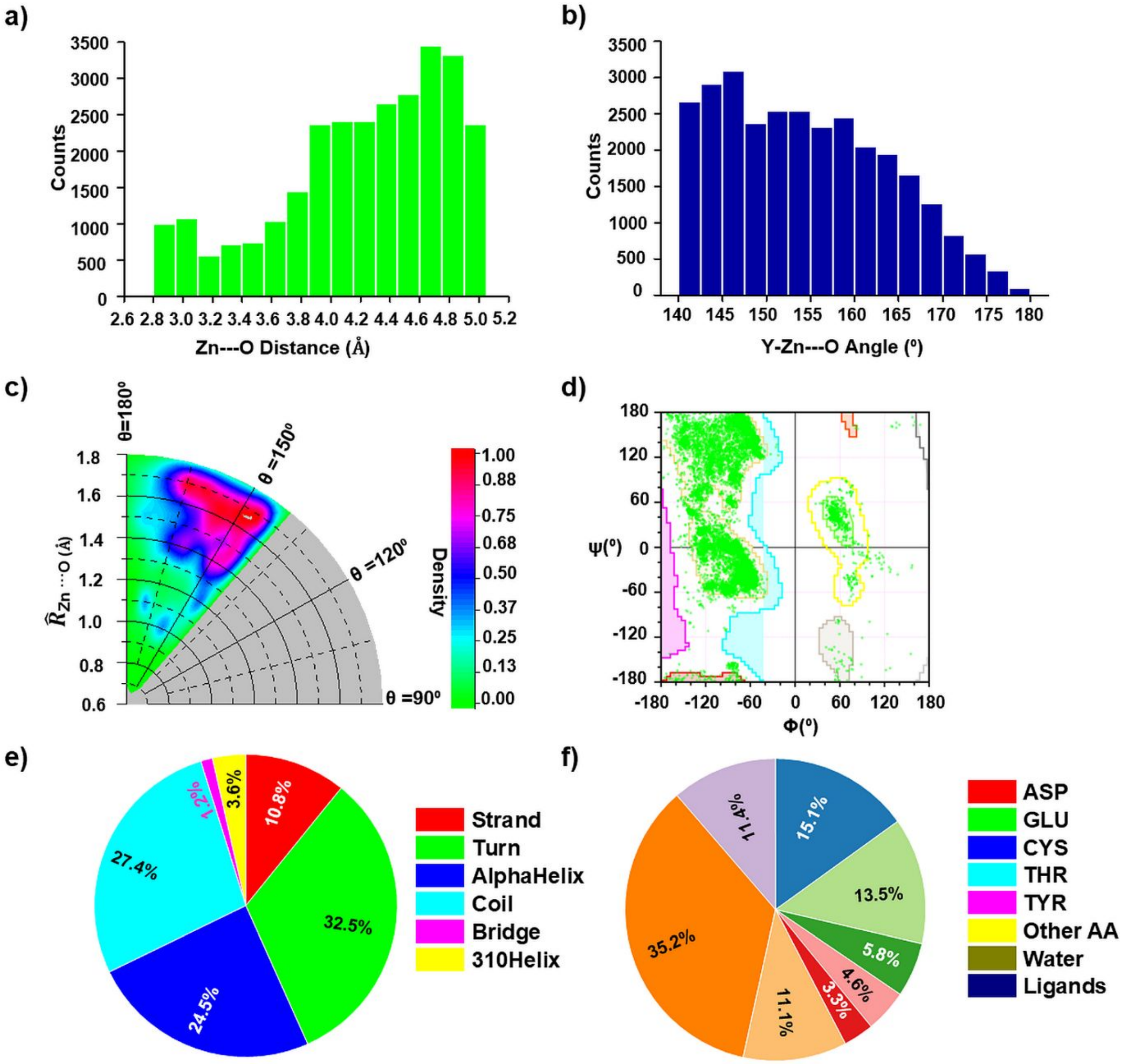

Figure S1 Statistical data of Zn···O contacts. Histogram distribution of a) the distance between Zn and O (Zn···O) and b) Y-Zn···O angle in proteins. c) Radial distribution of Zn···O spodium bonds in proteins. The angle (θ) made by O with respect to Zn-Y bond is plotted against the distance of Zn to O ($\hat{R}_{Zn\cdots O}$) atom, in which the sum of van der Waals radii of Zn and O atom has been taken as the normalization factor. d) Ramachandran plot of oxygen atom belonging to the amino acid residue showing that interacting residues are present in the favorable secondary structure regions. e) Pie chart for secondary structure distribution of the residues participating in the spodium bonds through O donors. About 32.5% of them are located in the turn followed by coil (27.4%), alpha helix (24.5%), and strand (10.8%). f) Pie chart for O donors involved in spodium bond. Water molecules are the prominent spodium bond donor (35.2%) followed by ASP (15.1%), GLU (13.5%) and non-amino acid ligands (11.4%).

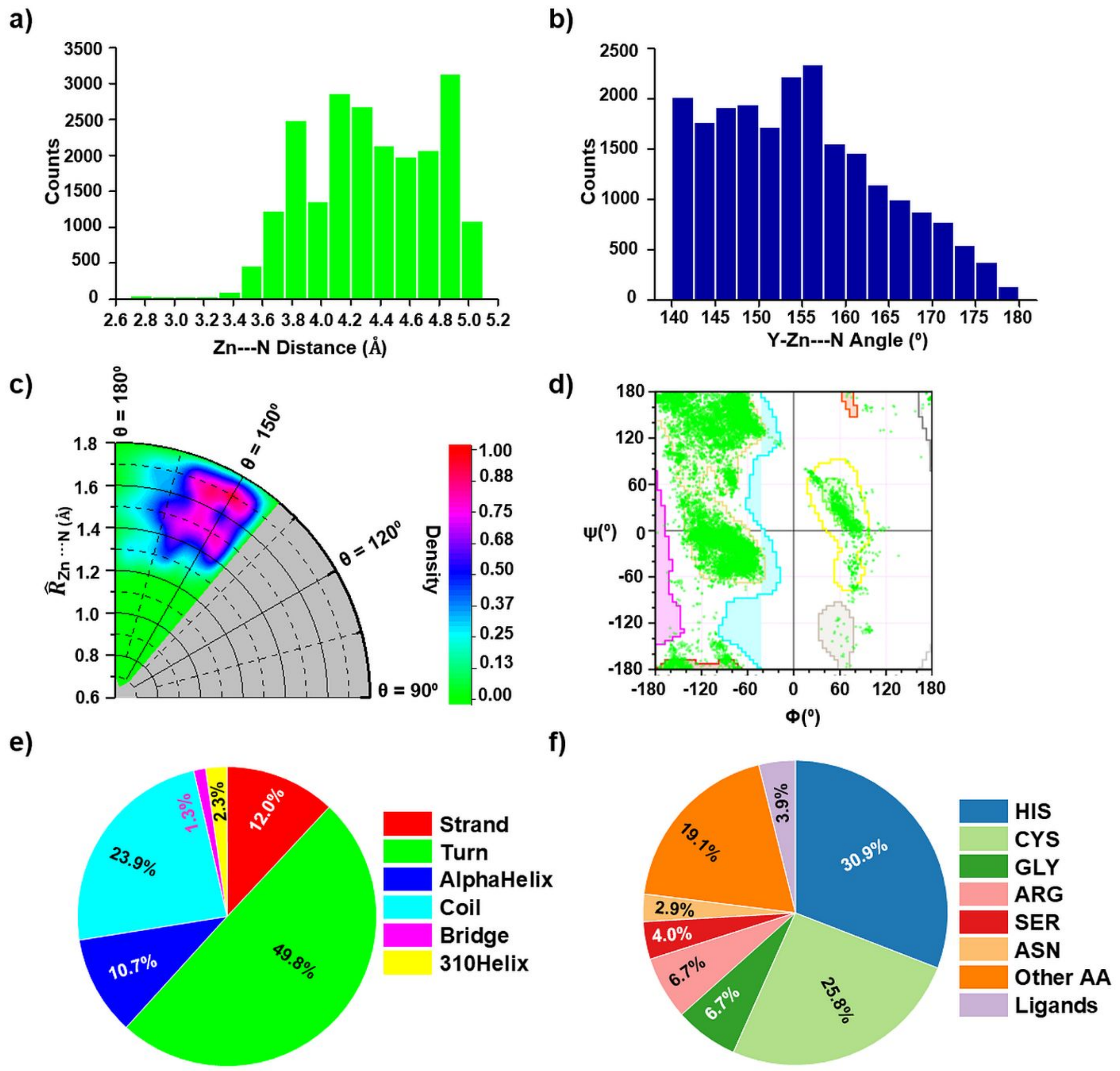

Figure S2 Statistical data of Zn···N contacts. Histogram distribution of a) the distance between Zn and N (Zn···N) and b) Y-Zn···N angle in proteins. c) Radial distribution of Zn···N spodium bonds in proteins. The angle (θ) made by N with respect to Zn-Y bond is plotted against the distance of Zn to N ($\hat{R}_{Zn\cdots N}$) atom, in which the sum of van der Waals radii of Zn and N atom has been taken as the normalization factor. d) Ramachandran plot of nitrogen atom belonging to the amino acid residue showing that interacting residues are present in the favorable secondary structure regions. e) Pie chart for secondary structure distribution of the residues participating in the spodium bonds through N donors. About 49.8% of them are located in the turn followed by coil (23.9%), strand (12%), and alpha helix (10.7%). f) Pie chart for N donors involved in spodium bond. N from HIS are the prominent spodium bond donor (30.9%) followed by CYS (25.8%). The non-amino acid ligands involvement in the spodium bond formation through N is rare (3.9%).

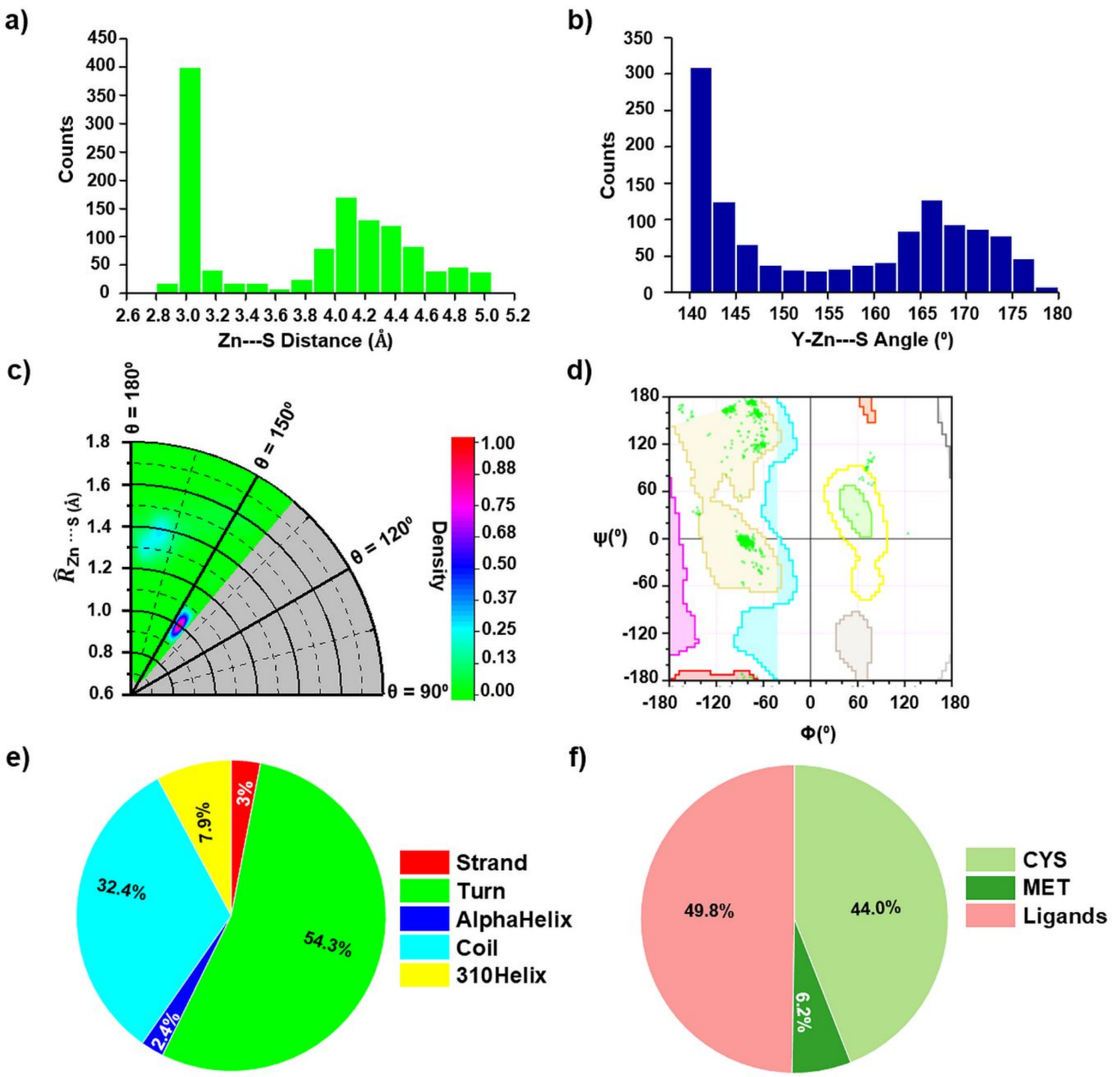

Figure S3 Statistical data of Zn···S contacts. Histogram distribution of a) the distance between Zn and S (Zn···S) that follows Gaussian distribution pattern centered around 3 Å and the 4.1 Å and b) Y-Zn···S angle in proteins. c) Radial distribution of Zn···S spodium bonds in proteins. The angle (θ) made by S with respect to Zn-Y bond is plotted against the distance of Zn to S ($\hat{R}_{Zn\cdots S}$) atom, in which the sum of van der Waals radii of Zn and S atom has been taken as the normalization factor. d) Ramachandran plot of oxygen atom belonging to the amino acid residue showing that interacting residues are present in the favorable secondary structure regions. e) Pie chart for secondary structure distribution of the residues participating in the spodium bonds through S donors. About 54.3% of them are located in the turn followed by coil (32.4%), and 3_{10} helix (7.9%). f) Pie chart for S donors involved in spodium bond. Both CYS and MET sulfur atoms participate almost equally in the formation of spodium bonds.

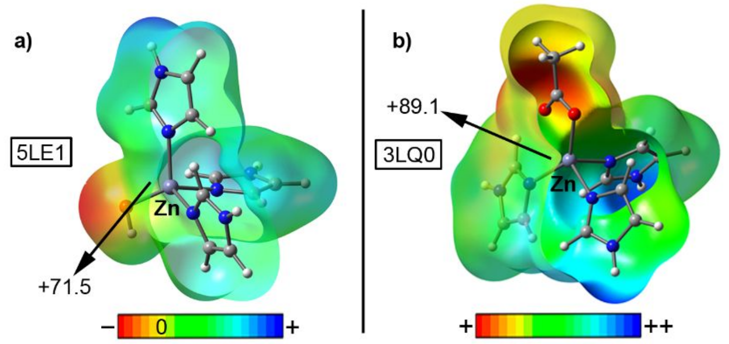

Figure S4. MEP (Molecular Electrostatic Potential) surfaces for Zn(II) centers present in 5LE1 (a) and 3LQ0 (b) structures with indication of the energy values at concrete regions of the surface in kcal·mol^{-1} (0.002 a.u.).

Cartesian Coordinates of the PDB models

2ZNR

C	-0.0866022	0.7500994	4.0776611
N	0.4143978	1.9150994	3.5466611
C	0.2143978	-0.2249006	3.1896611
C	0.9703978	1.6640994	2.3736611
N	0.8613978	0.3720994	2.1306611
C	3.3293978	1.8800994	-0.5653389
S	1.8733978	1.0840994	-1.2263389
C	-2.0346022	2.3470994	-2.1243389
C	-2.8046022	2.1590994	-0.8353389
O	-2.2226022	2.0500994	0.2526611
C	-1.6376022	0.9920994	-2.6843389
N	-4.1266022	2.1150994	-0.9873389
C	-5.0616022	1.9310994	0.1046611
C	-1.1386022	-2.7859006	-1.8503389
N	-2.0296022	-2.0639006	-1.0933389
C	0.0923978	-2.5149006	-1.3463389
C	-1.3706022	-1.3649006	-0.1843389
N	-0.0806022	-1.6259006	-0.3123389
C	4.0113978	-3.7949006	0.4636611
N	4.8243978	-2.7579006	0.0616611
C	2.8243978	-3.2319006	0.8056611
C	4.1563978	-1.6209006	0.1386611
N	2.9393978	-1.8789006	0.5896611
Zn	1.4433978	-0.4899006	0.3816611
H	0.3204420	2.8400324	3.9725459
H	0.0111703	-1.2994976	3.2186683
H	1.3750535	2.4027787	1.6726303
H	4.2794832	1.4120524	-0.9129888
H	3.3734673	1.9067024	0.5455825
H	-2.6214392	2.9283098	-2.8681097
H	-0.9558988	0.4766347	-1.9791100
H	-2.5213797	0.3435848	-2.8650222
H	-4.5097087	2.2728316	-1.9205775
H	-5.7326908	2.8076435	0.2196641
H	-4.4685026	1.8176327	1.0317087
H	-5.6911321	1.0275979	-0.0394553
H	1.0831507	-2.8617210	-1.6542284
H	-1.8056686	-0.6114442	0.4857522
H	5.7911654	-2.8625058	-0.2556908
H	1.8988318	-3.6881334	1.1691246
H	4.5312444	-0.6340234	-0.1531912
H	3.3738012	2.9345647	-0.9099804
H	-0.5932217	0.7294045	5.0475373
H	-1.4683526	-3.4349380	-2.6674359
H	4.3651650	-4.8304718	0.4816872
H	-1.0893882	1.0898263	-3.6421427
H	-3.0397376	-2.0359578	-1.2460319
H	-1.1221787	2.9262233	-1.8808642

3F1A

C	3.2492539	-1.0896421	-1.9270043
N	2.9012539	-2.0296421	-0.9820043
C	2.1412539	-0.3416421	-2.1440043
C	1.6362539	-1.8556421	-0.6410043
N	1.1542539	-0.8356421	-1.3270043
C	1.1132539	3.6513579	0.6979957
C	0.7842539	2.1703579	0.5689957
O	-0.1047461	1.8743579	-0.2550043

O	1.3782539	1.3063579	1.2529957
C	-0.1907461	-0.5216421	4.0349957
C	0.6742539	-1.5786421	3.3459957
O	0.3072539	-2.0986421	2.2939957
N	1.8252539	-1.8996421	3.9379957
C	2.6792539	-2.9516421	3.3729957
C	-4.1447461	-1.0376421	0.3369957
N	-4.0037461	0.3033579	0.6299957
C	-2.9677461	-1.4316421	-0.2020043
C	-2.7887461	0.6963579	0.2959957
N	-2.1377461	-0.3396421	-0.2090043
C	-1.7187461	0.4703579	-3.9690043
N	-1.0357461	1.0683579	-2.9340043
C	-1.8217461	1.4053579	-4.9430043
C	-0.7377461	2.3103579	-3.2650043
N	-1.2037461	2.5433579	-4.4800043
Zn	-0.5347461	0.2463579	-1.0950043
H	3.5298401	-2.7331431	-0.5895058
H	1.9762629	0.5085862	-2.8117058
H	1.1233915	-2.3593877	0.1939295
H	0.1967790	4.2354781	0.9210574
H	1.5098792	4.0338524	-0.2658407
H	0.0752304	-0.3277880	5.0927444
H	2.1139285	-1.4321971	4.7971436
H	2.0634815	-3.8348124	3.1114801
H	3.4472316	-3.2412119	4.1134298
H	3.1895537	-2.6020699	2.4489664
H	-2.6477044	-2.4133049	-0.5627490
H	-2.3661008	1.7023896	0.4118140
H	-2.2813105	1.3720847	-5.9364617
H	-0.2216646	3.0166250	-2.6037620
H	-1.1267608	3.4217520	-4.9979587
H	-4.7205498	0.8854411	1.0693893
H	-5.0708057	-1.5810315	0.5484342
H	-2.0697708	-0.5647426	-3.9301957
H	4.2538891	-1.0504197	-2.3589123
H	1.8637476	3.8156925	1.4907980
H	-0.0728743	0.4154331	3.4513301
H	-1.2462436	-0.8442462	3.9700564

5LE1

C	-1.82367360	-1.24286030	5.84151590
N	-2.79467360	-0.53886030	5.16451590
C	-0.83767360	-1.47986030	4.93551590
C	-2.39467360	-0.31686030	3.92151590
N	-1.20267360	-0.87286030	3.75351590
C	2.28032640	-1.29086030	3.30751590
N	1.41432640	-1.94486030	2.45451590
C	3.16032640	-2.22386030	3.74351590
C	1.76232640	-3.21686030	2.37551590
N	2.81732640	-3.41386030	3.14851590
C	-2.25406001	-4.87661422	1.24791478
N	-3.09994527	-4.04204135	0.55405654
C	-1.23392227	-4.09938480	1.68269106
C	-2.60674974	-2.81371619	0.55823610
N	-1.46867360	-2.82186030	1.23451590
Zn	-0.40567360	-1.21886030	1.87451590
C	1.14232640	-0.50586030	-1.93848410
O	1.05932640	-1.67886030	-1.60848410
C	1.80632640	1.66413970	-1.87648410
C	4.32432640	0.27613970	-0.28848410

C	2.81332640	0.04213970	-0.14048410
C	4.91832640	1.47913970	0.02551590
C	6.28832640	1.68513970	-0.07248410
C	-0.73067360	1.84713970	-4.89348410
C	0.19132640	2.30513970	-3.97948410
C	0.72932640	1.43713970	-3.05648410
C	0.35932640	0.07013970	-3.03048410
C	-0.56367360	-0.38686030	-3.96048410
C	-1.09467360	0.48113970	-4.89848410
C	-0.99267360	-1.83786030	-4.00448410
O	-1.54167360	-2.35586030	-3.01048410
O	-0.81867360	-2.49686030	-5.07948410
N	1.98532640	0.33813970	-1.28248410
C	7.09432640	0.64713970	-0.47648410
C	6.52532640	-0.57286030	-0.78548410
C	5.15432640	-0.75086030	-0.68448410
F	4.60332640	-1.94086030	-1.00148410
Cl	3.92632640	2.79813970	0.55951590
O	-0.48667360	0.31213970	0.72451590
H	-3.65843740	-0.17563100	5.57377200
H	0.11424860	-2.00781040	5.04875660
H	-2.92791960	0.28645260	3.14065680
H	4.01907550	-2.15084300	4.41889030
H	1.25285420	-3.97881670	1.77641620
H	3.32122440	-4.29522820	3.26857960
H	-3.97388655	-4.32079367	0.10376652
H	-0.35603196	-4.34950577	2.28607997
H	-3.05084692	-1.92650604	0.08397731
H	1.38985830	2.37701380	-1.12898650
H	2.75341350	2.09152230	-2.27038310
H	2.43112910	0.63933410	0.71607440
H	2.62010980	-1.02649870	0.07600200
H	6.70390630	2.66957860	0.18306810
H	-1.17406370	2.52802600	-5.63431900
H	0.49402020	3.36483590	-3.98749280
H	-1.80616220	0.10800840	-5.64931790
H	8.18227400	0.78496600	-0.56079160
H	7.13871110	-1.42425230	-1.11619420
H	2.18267070	-0.22063000	3.52037310
H	-1.93527260	-1.52285730	6.89360200
H	-2.47134821	-5.94084419	1.37979065
H	-0.02865580	1.08520950	1.20340950
H	-1.18444680	-3.40235880	-4.95189610

3LQ0

C	2.5495906	3.7991661	4.4248077
C	2.6445906	3.0421661	3.1088077
O	2.6845906	3.4801661	1.9288077
O	2.7165906	1.8271661	3.2218077
C	3.2635906	-2.8248339	1.7798077
C	3.2485906	-1.5488339	2.2128077
N	2.5655906	-2.8398339	0.5938077
C	2.1405906	-1.6228339	0.3258077
N	2.5955906	-0.8138339	1.2578077
C	6.9635906	1.1591661	0.5698077
C	5.9035906	1.0651661	1.3998077
N	6.4565906	1.5081661	-0.6711923
C	5.1415906	1.6021661	-0.5901923
N	4.7845906	1.3461661	0.6498077
C	1.4155906	2.2231661	-2.6211923
C	1.5305906	1.3481661	-1.5991923

N	1.7815906	3.4521661	-2.1171923
C	2.0485906	3.3291661	-0.8351923
N	1.8835906	2.0701661	-0.4931923
C	-1.6444094	-3.0608339	-3.8781923
C	-2.4544094	-3.0328339	-2.5951923
O	-2.3984094	-3.9458339	-1.7631923
N	-3.2594094	-1.9868339	-2.4271923
C	-4.1834094	-1.8868339	-1.2751923
C	-5.5334094	-2.5678339	-1.5331923
O	-5.8414094	-3.0218339	-2.6341923
C	-4.3484094	-0.4198339	-0.8881923
C	-3.1984094	0.1521661	-0.0771923
C	-2.0914094	-0.6198339	0.2368077
C	-3.2714094	1.4351661	0.4518077
C	-1.0794094	-0.1418339	1.0368077
C	-2.2224094	1.9391661	1.2528077
C	-1.1164094	1.1201661	1.4908077
O	-0.0664094	1.5481661	2.2688077
N	-6.3034094	-2.6328339	-0.4591923
C	-7.6054094	-3.3178339	-0.4951923
Zn	2.7565906	1.2971661	1.2548077
H	-1.4118877	-2.0537367	-4.2800009
H	-5.2846087	-0.2912512	-0.3051409
H	-4.5179918	0.1828672	-1.8091789
H	-2.0086530	-1.6521317	-0.1331300
H	-4.1518995	2.0684381	0.2575468
H	-0.2207864	-0.7726449	1.3077599
H	-2.2753609	2.9542314	1.6763011
H	-0.1263065	2.5104090	2.4139706
H	-3.7062592	-2.4701204	-0.4620279
H	-7.5765937	-4.2643811	0.0833250
H	-7.8229271	-3.5515157	-1.5528188
H	3.4579624	3.5792062	5.0179022
H	1.6851504	3.4057430	4.9932190
H	3.6715573	-1.0811813	3.1071619
H	2.3430853	-3.6844227	0.0607639
H	1.5370590	-1.3535019	-0.5442420
H	5.8416851	0.8343926	2.4672011
H	7.0247999	1.6589393	-1.5077415
H	4.4680124	1.8501748	-1.4174880
H	1.3104477	0.2805483	-1.5723322
H	1.7778214	4.3273408	-2.6464093
H	2.3514712	4.1122431	-0.1274857
H	3.6729030	-3.7427091	2.2136266
H	1.1301544	2.0955154	-3.6704182
H	8.0389098	1.0322861	0.7309212
H	-2.2167813	-3.6165866	-4.6495333
H	-0.7086959	-3.6201481	-3.6953356
H	-8.4043041	-2.6681718	-0.0848669
H	2.4511955	4.8920656	4.2902019
H	-5.9421462	-2.3390691	0.4494751
H	-3.4680971	-1.3766747	-3.2197293

5UUD

C	0.6430724	1.7415616	-3.2549513
C	1.2080724	0.9085616	-2.3489513
N	-0.7149276	1.6515616	-3.0489513
C	-0.9619276	0.8205616	-2.0559513
N	0.1920724	0.3545616	-1.6109513
H	-1.3119276	2.0825616	-3.4939513
H	2.1180724	0.7515616	-2.2369513

H	-1.8029276	0.5975616	-1.7279513
C	3.0040724	1.8895616	2.0940487
C	2.7950724	0.9225616	1.1670487
N	1.8160724	2.0195616	2.7810487
C	0.9200724	1.1925616	2.2690487
N	1.4920724	0.5095616	1.2950487
H	1.6730724	2.5675616	3.4280487
H	3.4160724	0.6055616	0.5510487
H	0.0400724	1.0975616	2.5570487
C	-3.2529276	-1.0534384	2.0380487
C	-2.2459276	-1.0984384	0.9280487
O	-2.6599276	-1.0964384	-0.2559513
O	-1.0539276	-1.0504384	1.2390487
H	-2.7809276	-0.9994384	2.8830487
H	-3.7949276	-1.8574384	1.9980487
Zn	0.4920724	-0.7394384	0.0780487
O	-1.0329276	-2.6464384	-1.9789513
O	1.1480724	-2.4734384	-0.1329513
H	3.8873723	2.4828869	2.3503881
H	1.0704251	2.3733928	-4.0395474
H	-3.9168256	-0.1822654	1.8852891
H	1.0261731	-3.0704694	0.6340078
H	0.4888860	-2.8122816	-0.8395770
H	-1.6655337	-2.0884625	-1.4249434
H	-1.5265833	-3.4744898	-2.1300064

5A0X

C	0.6430724	1.7415616	-3.2549513
C	1.2080724	0.9085616	-2.3489513
N	-0.7149276	1.6515616	-3.0489513
C	-0.9619276	0.8205616	-2.0559513
N	0.1920724	0.3545616	-1.6109513
H	-1.3119276	2.0825616	-3.4939513
H	2.1180724	0.7515616	-2.2369513
H	-1.8029276	0.5975616	-1.7279513
C	3.0040724	1.8895616	2.0940487
C	2.7950724	0.9225616	1.1670487
N	1.8160724	2.0195616	2.7810487
C	0.9200724	1.1925616	2.2690487
N	1.4920724	0.5095616	1.2950487
H	1.6730724	2.5675616	3.4280487
H	3.4160724	0.6055616	0.5510487
H	0.0400724	1.0975616	2.5570487
C	-3.2529276	-1.0534384	2.0380487
C	-2.2459276	-1.0984384	0.9280487
O	-2.6599276	-1.0964384	-0.2559513
O	-1.0539276	-1.0504384	1.2390487
H	-2.7809276	-0.9994384	2.8830487
H	-3.7949276	-1.8574384	1.9980487
Zn	0.4920724	-0.7394384	0.0780487
O	-1.0329276	-2.6464384	-1.9789513
O	1.1480724	-2.4734384	-0.1329513
H	3.8873723	2.4828869	2.3503881
H	1.0704251	2.3733928	-4.0395474
H	-3.9168256	-0.1822654	1.8852891
H	1.0261731	-3.0704694	0.6340078
H	0.4888860	-2.8122816	-0.8395770
H	-1.6655337	-2.0884625	-1.4249434
H	-1.5265833	-3.4744898	-2.1300064

6SJ4

C	-5.6579523	1.0809779	0.4179373
C	-4.4519523	1.0289779	1.0249373
N	-5.4969523	0.4349779	-0.7400627
C	-4.2269523	0.0489779	-0.8700627
N	-3.5839523	0.3919779	0.1979373
C	-0.0359523	3.8349779	1.8329373
C	-0.6149523	3.1039779	0.8529373
N	-0.0579523	3.0599779	2.9769373
C	-0.6049523	1.9049779	2.6989373
N	-0.9839523	1.9099779	1.4069373
C	-1.1149523	-3.4070221	1.8969373
C	-1.5519523	-2.6530221	0.8599373
N	-0.7809523	-2.5530221	2.8829373
C	-0.9569523	-1.3210221	2.4579373
N	-1.4329523	-1.3550221	1.2239373
C	-2.5239523	2.3669779	-3.3550627
C	-1.7419523	1.2779779	-2.6300627
O	-1.4199523	1.4529779	-1.4660627
O	-1.5139523	0.2079779	-3.2180627
O	5.7210477	3.7319779	-1.1080627
C	5.0350477	2.7749779	-1.5070627
O	4.9250477	2.1799779	-2.5850627
C	4.2080477	2.2739779	-0.4590627
C	3.4440477	1.1289779	-0.5740627
C	2.6910477	0.6929779	0.5209373
O	1.9510477	-0.4650221	0.4819373
C	2.6340477	1.3679779	1.7289373
C	3.3760477	2.5239779	1.8359373
C	4.1260477	2.9429779	0.7599373
C	1.3050477	-0.9290221	-0.7000627
O	0.5510477	-0.2490221	-1.4020627
C	2.0280477	-3.2600221	0.0829373
O	1.1270477	-4.8840221	-3.6430627
C	1.5400477	-2.4350221	-0.9510627
C	1.2270477	-2.9990221	-2.1820627
C	1.4380477	-4.3610221	-2.3890627
C	1.9450477	-5.1680221	-1.3620627
C	2.2160477	-4.6250221	-0.1240627
Zn	-1.5769523	0.3349779	0.2049373
H	-4.1335943	1.4420706	1.9873769
H	-6.2160720	0.3127233	-1.4564849
H	-3.7855112	-0.4090378	-1.7679585
H	-0.7695236	3.2777676	-0.2180344
H	0.3612807	3.3334332	3.8672559
H	-0.8391834	1.1588666	3.4583696
H	-1.8790994	-2.9535875	-0.1400609
H	-0.3871608	-2.8199672	3.7868391
H	-0.6400038	-0.4390972	3.0060975
H	-3.3869159	1.8988684	-3.8676738
H	-1.8942531	2.8202170	-4.1478260
H	3.4557194	0.5814768	-1.5303727
H	2.0260748	0.9511128	2.5476225
H	3.3787716	3.1053451	2.7747081
H	4.7438342	3.8512910	0.8297380
H	2.2826214	-2.7979963	1.0474200
H	1.3983866	-5.8206637	-3.6524075
H	0.8146336	-2.3694890	-2.9849080
H	2.1091872	-6.2428291	-1.5502018
H	2.6085897	-5.2598922	0.6855002
H	-6.6173581	1.5000015	0.7355188
H	-1.0273544	-4.4918299	2.0060601

H	0.4430681	4.8182246	1.8189137
H	-2.8732579	3.1716979	-2.6771829

4P4F

C	-1.9661017	-0.6759378	3.7288055
C	-0.8201017	0.0350622	3.5848055
N	-1.8171017	-1.7979378	2.9388055
C	-0.6391017	-1.7739378	2.3518055
N	-0.0191017	-0.6679378	2.7178055
C	4.9618983	-3.0949378	2.0718055
C	3.9538983	-2.1399378	1.4798055
O	2.9848983	-1.7829378	2.1878055
O	4.1138983	-1.7619378	0.2978055
C	-4.4941017	0.9910622	0.2618055
C	-3.7451017	0.8070622	-1.0541945
O	-3.4271017	1.8030622	-1.7631945
N	-3.4471017	-0.4449378	-1.3901945
C	-2.5211017	-0.6989378	-2.5411945
C	-3.1661017	-0.3449378	-3.8701945
O	-2.4771017	-0.1769378	-4.8661945
C	-2.0361017	-2.1649378	-2.5691945
C	-1.0861017	-2.5259378	-1.3901945
C	0.2978983	-1.8459378	-1.4601945
O	0.5438983	-1.0369378	-2.3721945
O	1.1308983	-2.1239378	-0.5791945
N	-4.4921017	-0.2039378	-3.8741945
C	-5.2011017	0.1300622	-5.0891945
C	2.5258983	1.9300622	5.8308055
C	2.3588983	0.9020622	4.6838055
O	2.3028983	-0.2839378	4.9878055
O	2.3108983	1.2530622	3.4628055
Zn	1.8828983	-0.1069378	2.1878055
N	1.6058983	2.6580622	-1.1611945
C	1.1558983	2.1870622	-2.5171945
C	4.7278983	3.5020622	-0.8661945
O	3.8508983	2.6190622	0.1808055
O	3.3638983	0.7940622	-1.4701945
O	1.9458983	0.7940622	0.5998055
P	2.7458983	1.6690622	-0.3881945
H	-5.2652461	1.7589773	0.1268814
H	-2.9114565	-2.8416098	-2.6161272
H	-1.4660740	-2.2646856	-3.5023045
H	-1.5504578	-2.2613954	-0.4185032
H	-0.9319221	-3.6164900	-1.3609152
H	-1.6284704	-0.0619502	-2.4390564
H	-4.6529055	-0.2915784	-5.9409330
H	0.7989391	1.1463202	-2.5114309
H	0.7952704	2.8455149	-0.5702550
H	-0.5170722	0.9874415	4.0017115
H	-2.5166741	-2.5215358	2.8138613
H	-0.2280145	-2.4536790	1.5936868
H	5.6239863	-2.5487959	2.7651341
H	5.5713168	-3.5286435	1.2729085
H	2.1999272	2.9128404	5.4797461
H	3.5941265	1.9703671	6.0879535
H	-6.2172828	-0.2880615	-5.0591809
H	-5.2686409	1.2209974	-5.2408717
H	-4.9589603	0.0674565	0.6393699
H	-3.7820029	1.3583084	1.0150834
H	0.3664627	2.8711795	-2.8671031
H	-2.8727612	-0.4964525	4.2945840

H	2.0117702	2.2375428	-3.1999413
H	4.4779735	-3.8962382	2.6545804
H	1.9642804	1.6097269	6.7166432
H	-3.5768375	-1.1980267	-0.7212381
H	-4.9879613	-0.2426363	-2.9919679
H	3.2013643	-0.1758912	-1.2575561
H	5.2773721	2.7864784	-1.5026633
H	4.0017335	4.1149304	-1.4306958
H	5.3819844	4.0890169	-0.2027934

4G9L

C	-0.3165559	-4.9899173	-3.3451614
C	0.0744441	-3.7279173	-3.0591614
N	-0.0065559	-5.7529173	-2.2221614
C	0.5114441	-4.9629173	-1.2841614
N	0.5944441	-3.7389173	-1.7721614
C	-3.0725559	-0.6879173	-0.7351614
C	-1.8525559	-0.7769173	-1.3001614
N	-2.9405559	-1.1839173	0.5458386
C	-1.7075559	-1.6159173	0.7318386
N	-1.0225559	-1.3989173	-0.3771614
C	1.4144441	-3.1569173	3.2998386
C	1.0384441	-3.3819173	2.0168386
N	2.1844441	-2.0199173	3.2808386
C	2.3564441	-1.6329173	2.0198386
N	1.6494441	-2.4309173	1.2348386
C	-1.5345559	6.6210827	-0.7271614
C	-0.6425559	5.6090827	-0.0721614
O	0.3204441	5.9700827	0.6088386
N	-0.9415559	4.3430827	-0.2801614
C	-0.1075559	3.2540827	0.1988386
C	-1.0205559	2.2050827	0.8418386
C	-0.6885559	1.8980827	2.2718386
C	0.7834441	1.6110827	2.4678386
O	1.2714441	0.5940827	1.9218386
O	1.4444441	2.3970827	3.1668386
C	2.4884441	1.3150827	-1.8821614
C	2.0284441	-0.1329173	-1.8031614
O	2.0124441	-0.9119173	-2.7371614
O	1.5494441	-0.5449173	-0.7731614
Zn	0.8734441	-2.1539173	-0.6571614
H	-2.4595765	6.2102951	-1.1844706
H	-2.0830997	2.5224227	0.7394506
H	-0.9139835	1.2568787	0.2696643
H	-0.9544194	2.7537999	2.9279898
H	-1.2870370	1.0183831	2.6103011
H	0.5998057	3.6866274	0.9356625
H	3.4091697	1.3988473	-2.4902818
H	0.0899109	-2.8015382	-3.6429709
H	-0.1679241	-6.7583333	-2.1374676
H	0.8600199	-5.2901511	-0.2990507
H	-1.4863438	-0.4889936	-2.2901517
H	-3.6779829	-1.1615222	1.2529412
H	-1.2966119	-2.0230678	1.6631126
H	0.3961852	-4.1704146	1.6098964
H	2.5401891	-1.5064591	4.0889468
H	2.7829511	-0.6648792	1.7202406
H	2.6365065	1.7316014	-0.8692257
H	1.7009166	1.9001526	-2.3993042
H	-1.8154160	7.3860525	0.0224644
H	-1.7215250	4.1054950	-0.8943431

H	1.1573929	-3.6620406	4.2366935
H	-4.0143118	-0.2516743	-1.0825185
H	-0.7508098	-5.4418465	-4.2419604
H	-0.9587383	7.1473830	-1.5157127
H	0.4939484	2.8247107	-0.6274337

1UXM

C	1.9944420	-0.1757713	-4.2340884
C	2.4314420	-1.3957713	-4.6340884
N	0.7734420	-0.3797713	-3.6190884
C	0.4834420	-1.6707713	-3.6580884
N	1.4664420	-2.3087713	-4.2850884
C	-2.7325580	0.4742287	-1.1620884
C	-3.8205580	-0.3327713	-1.2440884
N	-2.0305580	0.2862287	-2.3330884
C	-2.6595580	-0.6027713	-3.0830884
N	-3.7495580	-0.9877713	-2.4450884
C	-0.7057499	3.7611455	-1.5825089
C	-0.8828184	4.1298220	-0.2625241
N	-0.0587863	2.5325948	-1.6222836
C	0.1573463	2.1711984	-0.3500668
N	-0.3305040	3.1085983	0.4964914
C	-3.0135580	2.7702287	-5.9960884
C	-2.1875580	2.5212287	-4.6760884
O	-1.0075580	2.1122287	-4.6290884
O	-2.6765580	2.7962287	-3.5610884
C	-2.6065580	0.3762287	4.1289116
C	-1.3365580	0.5432287	3.3179116
O	-0.9525580	1.6672287	2.9359116
N	-0.6865580	-0.5867713	3.0589116
C	0.5574420	-0.5687713	2.3399116
C	0.4414420	-1.2647713	0.9749116
O	0.7204420	-0.6337713	-0.0420884
C	1.6674420	-1.1457713	3.2029116
C	2.2224420	-0.1247713	4.1949116
C	3.3204420	-0.7537713	5.0119116
C	2.7694420	-1.9927713	5.7039116
N	3.3884420	-2.1997713	7.0399116
N	-0.0105580	-2.5247713	0.9649116
C	-0.1265580	-3.3177713	-0.2410884
Zn	-0.4615580	1.1982287	-3.1260884
H	-3.4530202	0.8046232	3.5568369
H	1.2939344	-2.0567096	3.7170686
H	2.5002205	-1.4887508	2.5490393
H	1.4047071	0.2366348	4.8579206
H	2.5817912	0.7641256	3.6336334
H	3.6914209	-0.0410061	5.7785253
H	4.1999019	-1.0055600	4.3824431
H	1.6505161	-1.9030123	5.7669606
H	2.9651568	-2.9056996	5.1010699
H	3.0516264	-3.0773599	7.4553977
H	3.0837067	-1.4510039	7.6764024
H	0.7648541	0.4915796	2.1120160
H	-0.9926248	-1.4461964	3.5170484
H	0.5069695	-2.8519594	-1.0197106
H	-0.1860613	-2.9897173	1.8566792
H	3.3420739	-1.7020838	-5.1592160
H	-0.4107609	-2.1389665	-3.2366400
H	1.5237881	-3.3150725	-4.4539178
H	-4.6587983	-0.4927480	-0.5582591
H	-2.3776104	-0.8979327	-4.0995499

H	-4.4621918	-1.6286524	-2.8006661
H	-1.3510172	5.0094533	0.1883434
H	0.6075562	1.2175920	-0.0457388
H	-0.4153889	2.9477294	1.5134046
H	-4.0693516	2.9171523	-5.7138049
H	-2.6296439	3.6849377	-6.4908706
H	-2.8963108	1.9295644	-6.7082675
H	-1.0608582	4.2445247	-2.4959092
H	0.2185320	-4.3545251	-0.0608971
H	-1.1732542	-3.3522107	-0.6157326
H	-2.5210056	0.9748976	5.0571975
H	-2.8448791	-0.6720909	4.3952125
H	-2.4030918	1.1821022	-0.3962520
H	2.4428320	0.8169111	-4.3332979

1F18

C	-0.4828986	-0.6692555	3.2972056
N	-0.1548986	-0.8792555	1.9752056
C	-1.2158986	-1.7442555	3.6842056
C	-0.6778986	-2.0252555	1.5662056
N	-1.3268986	-2.5682555	2.5852056
C	0.6151014	-0.3272555	-2.3027944
N	1.0201014	-0.7132555	-1.0327944
C	0.8351014	-1.3782555	-3.1177944
C	1.4741014	-1.9582555	-1.0987944
N	1.3761014	-2.3772555	-2.3497944
C	1.7391014	3.1497445	1.3502056
N	0.7791014	2.2067445	1.0392056
C	1.2621014	4.3327445	0.8982056
C	-0.2348986	2.7937445	0.4292056
N	0.0341014	4.0857445	0.3342056
C	5.3921014	-0.6302555	0.5832056
C	4.0081014	-0.0272555	0.4022056
O	3.1311014	-0.4092555	1.1982056
O	3.7811014	0.7957445	-0.5247944
Zn	1.1591014	0.0467445	0.7972056
H	-1.6855048	-1.9985542	4.6396553
H	-0.5983176	-2.4472524	0.5605096
H	-1.8371636	-3.4541428	2.5730635
H	0.6814866	-1.5044595	-4.1945654
H	1.9535389	-2.4997386	-0.2740549
H	1.6836353	5.3429355	0.9270528
H	-1.1698636	2.2947052	0.1251079
H	-0.5797524	4.8011837	-0.0602466
H	6.1662695	0.0889136	0.2580975
H	5.4806220	-1.5273480	-0.0658792
C	-3.9048986	1.7327445	-1.8717944
C	-3.3678986	0.5597445	-1.0787944
O	-2.7848986	0.7437445	0.0142056
N	-3.6298986	-0.6462555	-1.6007944
C	-3.2558986	-1.8942555	-0.9187944
H	-3.0753076	2.4307068	-2.1003916
H	-4.1277321	-0.7076896	-2.4892805
H	-4.0819896	-2.6293553	-0.9787180
H	-3.0540409	-1.6417055	0.1390434
H	-2.3419133	-2.3433979	-1.3663817
H	5.5697341	-0.9493223	1.6276645
H	0.2128497	0.6687971	-2.5043807
H	-0.1545545	0.2183287	3.8449265
H	2.6935811	2.8801779	1.8097013
H	-4.4086702	1.4621801	-2.8204962

H	-4.6219530	2.2841052	-1.2319480
H	1.6996387	-3.2794821	-2.7066783

2VR7

C	-2.3916379	3.2740745	-0.6730813
N	-2.2786379	1.9110745	-0.8000813
C	-2.2556379	3.7820745	-1.9200813
C	-2.0846379	1.6010745	-2.0700813
N	-2.0766379	2.7230745	-2.7780813
C	-0.5676379	-2.0359255	-0.0090813
N	-1.6256379	-1.1559255	-0.1970813
C	-0.7656379	-3.0549255	-0.8720813
C	-2.4556379	-1.6799255	-1.0840813
N	-1.9496379	-2.8259255	-1.5250813
C	-1.7246379	1.4220745	3.5569187
N	-1.2586379	1.3020745	2.2649187
C	-0.6346379	1.4730745	4.3649187
C	0.0793621	1.3190745	2.2969187
N	0.4703621	1.4590745	3.5549187
C	-5.6636379	-1.5549255	1.8089187
C	-4.2936379	-0.9669255	1.7369187
O	-4.1176379	0.0510745	0.9939187
O	-3.3466379	-1.4349255	2.4139187
Zn	-2.2476379	0.5510745	0.6629187
H	-2.2842149	4.8128581	-2.2882116
H	-1.9431509	0.5933481	-2.4713194
H	-1.9604272	2.8029902	-3.7902196
H	-0.1852711	-3.9657625	-1.0536126
H	-3.4319326	-1.2576692	-1.3513774
H	-0.5492734	1.5633281	5.4524085
H	0.7391099	1.2967726	1.4128349
H	1.4407469	1.4756704	3.8756170
H	-5.9409039	-1.7183031	2.8681919
H	-5.6596237	-2.5571271	1.3319163
C	4.3943621	-3.2209255	0.4629187
C	4.0003621	-1.7889255	0.7189187
O	3.7323621	-1.4259255	1.8469187
N	4.0573621	-0.9619255	-0.3210813
C	3.6953621	0.4250745	-0.2450813
C	2.4193621	0.7350745	-0.9960813
O	1.5213621	1.4550745	-0.4640813
C	4.8593621	1.2410745	-0.8050813
N	2.2913621	0.2290745	-2.2190813
C	1.1773621	0.5680745	-3.0980813
H	3.6231525	-3.8812005	0.9042231
H	5.3357809	-3.4299410	1.0092410
H	4.5338781	-3.4930935	-0.6033498
H	4.4842156	-1.3067312	-1.1833188
H	3.5124693	0.6548005	0.8210958
H	5.7705945	1.0693553	-0.1999779
H	5.0806825	0.9553166	-1.8556376
H	3.0318194	-0.3634060	-2.5962067
H	0.6445317	1.4218446	-2.6399181
H	0.4691031	-0.2828131	-3.2011613
H	1.5469733	0.8487641	-4.1041418
H	4.6368260	2.3267533	-0.7987375
H	-6.4238919	-0.9248673	1.3110724
H	-2.5472076	3.7608853	0.2936653
H	-2.7906913	1.3686801	3.7939845
H	0.2418922	-1.8332395	0.6983642
H	-2.3942446	-3.4691769	-2.1833870

6R6F

C	-2.3610605	-0.6498051	-2.6905863
N	-2.5570605	0.5671949	-2.0195863
C	-1.7630605	-1.4928051	-1.8065863
C	-2.0800605	0.4731949	-0.7805863
N	-1.5790605	-0.8328051	-0.6395863
C	0.0289395	-5.6708051	-0.0015863
N	1.2599395	-5.0248051	-0.2415863
C	-0.8120605	-4.6598051	0.4134137
C	1.1389395	-3.7218051	0.0264137
N	-0.0740605	-3.4468051	0.4524137
C	-3.7020605	-2.1518051	2.3444137
N	-2.3000605	-2.3848051	2.3114137
C	-4.2660605	-2.9618051	3.3374137
C	-2.0000605	-3.3118051	3.1994137
N	-3.1660605	-3.6688051	3.8794137
Zn	-0.8440605	-1.6668051	1.0984137
O	1.0799395	2.8701949	-2.8855863
C	1.7889395	2.7221949	-1.8475863
N	2.9739395	2.1251949	-1.8295863
C	3.6459395	1.6601949	-3.1205863
C	1.2449395	3.0411949	-0.5315863
C	0.9089395	1.9941949	0.3744137
C	0.3089395	2.2231949	1.5874137
S	-0.1300605	0.8591949	2.6514137
O	-1.6430605	0.8321949	2.6114137
O	0.5229395	1.0761949	3.9674137
N	0.5079395	-0.4548051	1.9194137
C	0.7749395	4.3281949	-0.1875863
C	0.1789395	4.6161949	1.0264137
C	-0.1140605	3.5311949	1.8954137
Cl	-0.9620605	3.9281949	3.3554137
S	1.0859395	5.7661949	-1.2865863
C	2.8959395	5.8521949	-1.4095863
H	-2.9385289	1.4083765	-2.4441693
H	-1.4512969	-2.5226513	-1.9372215
H	-2.0715404	1.2226087	0.0042447
H	-1.8533559	-4.6909101	0.7075403
H	1.9280663	-2.9897962	-0.1141750
H	-5.2751446	-3.0828003	3.7095266
H	-1.0170350	-3.7051808	3.4289232
H	-3.2201326	-4.3442686	4.6353326
H	3.5299572	2.1609714	-0.9800836
H	4.2225973	0.7534881	-2.9093171
H	2.8499435	1.4599397	-3.8428565
H	1.1770364	0.9638452	0.1284158
H	1.3162476	-0.7527994	2.4670879
H	-0.1032215	5.6305598	1.2982946
H	3.3151935	5.0237107	-1.9961948
O	1.6069395	-0.9188051	-2.2865863
H	3.0958437	6.7917175	-1.9408755
H	3.3605349	5.8951377	-0.4167317
H	4.3039192	2.4481677	-3.5082497
H	-0.1105515	-6.7314451	-0.1669829
H	-4.1562372	-1.4138485	1.6965858
H	-2.6747112	-0.7737473	-3.7202344
H	2.0992765	-5.4780548	-0.5903219
H	0.7581872	-0.6668701	-2.6908486
H	1.9830841	-0.0644200	-2.0023528

4MDG

C	-1.72077380	3.61007250	-5.16665230
N	-1.89377380	4.78007250	-4.54265230
C	-1.03777380	2.78607250	-4.26765230
C	-1.41277380	4.71607250	-3.30465230
N	-0.85477380	3.49707250	-3.12965230
C	0.56222620	-1.36892750	-2.47865230
N	1.79022620	-0.85092750	-2.68965230
C	-0.23777380	-0.32192750	-2.09865230
C	1.82422620	0.45307250	-2.36565230
N	0.59422620	0.82307250	-2.04365230
C	-3.00677380	2.14807250	-0.16165230
N	-1.65677380	1.93007250	-0.17365230
C	-3.52677380	1.27507250	0.78634770
C	-1.32177380	1.03707250	0.73634770
N	-2.46577380	0.58007250	1.31134770
Zn	-0.14077380	2.57907250	-1.44365230
O	-0.13977380	5.65407250	-0.66565230
S	1.17222620	5.17207250	-0.33065230
O	1.57022620	5.38607250	1.02834770
N	1.23622620	3.56007250	-0.65765230
N	2.23722620	6.05007250	-1.23265230
C	2.06622620	6.07207250	-2.64165230
H	-0.69595130	1.75202790	-4.38231080
H	-1.42668080	5.51217160	-2.55161880
H	-1.30194540	-0.27077900	-1.85618280
H	2.74016690	1.04963580	-2.33645350
H	-4.55621260	1.10875020	1.11986740
H	-0.29688040	0.59435040	0.92354090
H	-2.50536120	-0.10310340	2.07101550
H	2.10092860	3.36695710	-1.20310970
H	1.00572320	6.28030830	-2.88080060
H	2.37281770	5.13767740	-3.17247740
H	2.64226260	-1.42160450	-2.74240080
C	-1.16877380	-6.52992750	0.87734770
C	0.05022620	-6.01692750	1.62734770
O	1.05522620	-6.70892750	1.82634770
N	-0.08777380	-4.78692750	2.08334770
C	0.94422620	-4.11392750	2.86934770
C	0.62022620	-4.24992750	4.34934770
O	1.38722620	-4.78192750	5.11734770
C	1.02722620	-2.62992750	2.46434770
C	1.46522620	-2.48692750	1.01334770
C	2.14322620	-1.18392750	0.70834770
O	1.57722620	-0.12992750	1.01134770
O	3.24322620	-1.21292750	0.18434770
N	-0.57277380	-3.78692750	4.74334770
C	-1.06777380	-4.14892750	6.08334770
H	-1.92515880	-5.75517950	0.63631210
H	-1.65455480	-7.31634930	1.48994590
H	-0.83787880	-7.01975710	-0.05894280
H	-0.89284450	-4.23434110	1.78627060
H	1.89940650	-4.64435600	2.70168070
H	0.06158310	-2.10879790	2.65653940
H	1.76242880	-2.13748480	3.13424840
H	0.58441780	-2.60831000	0.33965670
H	2.16561960	-3.30650110	0.75794700
H	-1.27064210	-3.55612390	4.03572080
H	-1.83077500	-3.41850160	6.41553140
H	-0.21157530	-4.13598440	6.78107240
H	-1.50491790	-5.17108870	6.10023330
H	-3.50895510	2.86594590	-0.81585370

H	0.34554890	-2.43175660	-2.62107690
H	-2.05120790	3.43076640	-6.19472440
H	2.66122050	6.90151430	-3.07241850
H	3.18767410	6.04111990	-0.83861980
H	-2.37340800	5.59410160	-4.93801470

6QNG

C	-1.87734620	-3.63343220	3.24063410
C	-1.65834620	-2.82943220	2.16663410
N	-0.81834620	-3.43043220	4.10263410
C	0.01765380	-2.54943220	3.57263410
N	-0.48834620	-2.13343220	2.40163410
C	-2.92334620	-0.79543220	-2.05636590
C	-1.87034620	-1.35543220	-1.43136590
N	-3.29934620	0.30956780	-1.31136590
C	-2.45134620	0.41956780	-0.29036590
N	-1.54334620	-0.60343220	-0.35536590
C	-1.68434620	-1.31443220	-8.48636590
C	-1.31734620	0.12156780	-8.08336590
O	-1.90534620	1.08956780	-8.60536590
N	-0.43734620	0.27856780	-7.08936590
C	0.08265380	1.59256780	-6.62736590
C	1.31765380	1.97056780	-7.46436590
O	1.33965380	3.06956780	-8.07336590
C	0.41265380	1.50256780	-5.13236590
C	-0.77834620	1.14556780	-4.23136590
C	-0.82834620	1.99356780	-2.96236590
O	-0.06734620	1.69856780	-1.99436590
O	-1.57834620	2.99856780	-2.96036590
N	2.31765380	1.10056780	-7.50536590
C	3.49165380	1.25556780	-8.41236590
C	2.01165380	-2.82543220	-0.39836590
C	2.68365380	-2.87943220	-1.56736590
N	1.33765380	-1.64743220	-0.38336590
C	1.56665380	-1.00643220	-1.51136590
N	2.36565380	-1.75043220	-2.26836590
Zn	0.03765380	-0.71343220	0.91863410
Cl	4.36665380	0.68556780	4.82263410
N	0.58365380	0.81456780	1.82363410
S	2.03465380	0.70856780	2.65863410
O	2.57265380	-0.58643220	2.47963410
O	2.85565380	1.76056780	2.35763410
C	1.63265380	0.79656780	4.27863410
C	2.66265380	0.72456780	5.35063410
C	2.37665380	0.77156780	6.74163410
C	1.07865380	0.84056780	7.20463410
C	0.02665380	0.87356780	6.15163410
C	0.33865380	0.85256780	4.75263410
N	0.68065380	0.83456780	8.53763410
C	1.26365380	0.42556780	9.81263410
C	-1.42434620	0.89256780	6.53563410
O	-1.82834620	0.04956780	7.34463410
N	-2.26334620	1.76056780	5.99363410
C	-3.69634620	1.69356780	6.28163410
H	-1.08397310	-2.10880360	-7.99590650
H	0.81474690	2.49640360	-4.84213270
H	1.26083980	0.79532440	-4.97769160
H	-0.75369290	0.06130460	-3.97432220
H	-1.71908900	1.32764760	-4.78945500
H	-0.68844680	2.35356160	-6.84451030
H	3.47325450	2.29919000	-8.77600440

H	-2.25582110	-2.67246430	1.26317130
H	-0.69207140	-3.90380190	5.00096760
H	0.95038930	-2.18457560	4.01835110
H	-1.29050280	-2.24061690	-1.71214340
H	-3.93977810	1.04521360	-1.62594600
H	-2.42850940	1.25993150	0.40860690
H	3.34833540	-3.64736160	-1.97615220
H	1.08962920	-0.02854440	-1.81307530
H	2.73232680	-1.48208070	-3.18368210
H	-0.13688520	1.23827450	2.43876420
H	3.23431720	0.73684530	7.42745030
H	-0.54593440	0.84683050	4.09377210
H	1.15846700	-0.66896150	9.98190530
H	-3.91212890	1.99301080	7.32815620
H	-4.23791300	2.36606610	5.59205270
H	3.41873620	0.56628350	-9.27914970
H	4.43309900	1.06229180	-7.86130580
H	-1.57005200	-1.40345330	-9.58383900
H	-2.75637690	-1.47272340	-8.25791840
H	-3.45338310	-1.07311190	-2.97197280
H	-2.68041640	-4.33938810	3.47451270
H	1.99714730	-3.52775660	0.44008020
H	2.33933430	0.68470670	9.87046250
H	0.74234050	0.96659020	10.62468600
H	-4.05553120	0.65458520	6.15387280
H	-1.89790760	2.55799240	5.46919330
H	-0.34241750	0.71699790	8.56928610
H	2.16877770	0.17170540	-7.10766120
H	-0.05424380	-0.55470570	-6.63822050

3MHC

C	-1.91315980	2.40021210	-4.73293410
C	-1.29215980	1.60321210	-3.83493410
N	-2.07815980	3.62321210	-4.12193410
C	-1.56915980	3.57121210	-2.90293410
N	-1.07315980	2.36221210	-2.70993410
C	0.52284020	-2.50978790	-2.17393410
C	-0.27515980	-1.51078790	-1.72193410
N	1.75384020	-1.94078790	-2.41993410
C	1.71084020	-0.65778790	-2.10393410
N	0.48884020	-0.37078790	-1.69493410
C	-1.17615980	-7.70478790	1.19206590
C	0.01784020	-7.16478790	1.96406590
O	1.03084020	-7.85078790	2.17606590
N	-0.10915980	-5.90778790	2.35606590
C	0.90284020	-5.23578790	3.15806590
C	0.57384020	-5.41578790	4.64206590
O	1.37384020	-5.95878790	5.39906590
C	0.94784020	-3.76078790	2.76106590
C	1.31084020	-3.58978790	1.28806590
C	2.01284020	-2.28478790	1.00406590
O	1.41684020	-1.22278790	1.26006590
O	3.16584020	-2.32978790	0.53406590
N	-0.61015980	-4.97378790	5.06306590
C	-1.06815980	-5.30678790	6.40106590
C	-3.13715980	0.94421210	0.25206590
C	-3.63015980	0.10021210	1.18506590
N	-1.77515980	0.72821210	0.21806590
C	-1.45615980	-0.18678790	1.11906590
N	-2.56115980	-0.57778790	1.72506590
Zn	-0.29015980	1.44621210	-1.09193410

C	1.05784020	8.95221210	-3.11993410
C	1.74684020	7.23821210	-1.96993410
C	1.30784020	5.27721210	-0.51893410
C	1.16784020	9.88621210	-4.46593410
N	1.06384020	2.67421210	-0.18593410
N	2.85684020	6.54621210	-1.50793410
N	2.65584020	5.38321210	-0.64993410
N	2.27884020	8.26221210	-2.70293410
O	-0.08415980	8.79421210	-2.85993410
O	1.12884020	4.16921210	1.67406590
O	-0.81115980	4.14221210	0.19506590
S	0.52884020	6.45921210	-1.35293410
S	0.53984020	3.90121210	0.29606590
H	-1.90521170	-6.93315610	0.86904850
H	1.70580440	-3.26027580	3.39900860
H	-0.01446270	-3.25330250	3.00350470
H	1.97765720	-4.41775490	0.97589980
H	0.39557960	-3.65800050	0.65649570
H	1.86867740	-5.74670510	2.98901150
H	-0.19383010	-5.30797880	7.07695420
H	-0.97107090	0.55918490	-3.90659650
H	-2.52766820	4.43973540	-4.54523230
H	-1.56235020	4.38668690	-2.16949390
H	-1.32425740	-1.51859530	-1.41560600
H	2.61666700	-2.47227380	-2.57204280
H	2.57411160	0.01372970	-2.12684540
H	-4.65287540	-0.05665370	1.54305020
H	-0.42286300	-0.62305160	1.28828040
H	-2.60765370	-1.26733560	2.47836250
H	2.18117300	10.26792780	-4.70123800
H	0.83131680	9.22442260	-5.28780760
H	0.45310110	10.71933690	-4.34942470
H	-2.26541490	2.21280870	-5.75222770
H	-3.64251280	1.66770260	-0.39331080
H	0.34499290	-3.57887090	-2.32183130
H	-1.70828740	-8.44529050	1.82356090
H	-0.80593890	-8.25246230	0.30459530
H	-1.52825520	-6.31867840	6.44773400
H	-1.80628650	-4.55910590	6.75215390
H	-1.31401200	-4.71443790	4.37105220
H	-0.93155580	-5.37472490	2.07169090
H	3.26169010	8.53863380	-2.61942240
H	1.90191610	2.79564600	-0.80041420

5JN8

C	4.12313770	2.89901180	-2.71899500
C	3.14713770	2.03301180	-2.35499500
N	4.48113770	3.58001180	-1.57499500
C	3.77513770	3.12001180	-0.55599500
N	2.96313770	2.17801180	-1.00299500
C	0.07113770	-1.71398820	-2.90799500
C	0.16813770	-0.47698820	-2.35799500
N	0.97513770	-2.50298820	-2.23299500
C	1.56813770	-1.78698820	-1.28999500
N	1.10213770	-0.55298820	-1.35099500
C	-6.22086230	-2.76098820	-3.97399500
C	-5.90886230	-3.49098820	-2.68099500
O	-6.16486230	-4.69198820	-2.55499500
N	-5.35586230	-2.74898820	-1.72299500
C	-5.05786230	-3.28298820	-0.40499500
C	-6.30386230	-3.18598820	0.47700500

O	-6.79586230	-4.20398820	0.99200500
C	-3.84586230	-2.56698820	0.20000500
C	-2.55386230	-2.90398820	-0.56199500
C	-1.32186230	-2.89898820	0.32300500
O	-1.01386230	-1.83698820	0.88900500
O	-0.66286230	-3.95198820	0.45400500
N	-6.80986230	-1.97098820	0.62500500
C	-8.10286230	-1.73998820	1.24800500
C	-0.77886230	3.20501180	-0.47799500
C	-2.12886230	3.21301180	-0.39599500
N	-0.37886230	1.94301180	-0.09199500
C	-1.44186230	1.22701180	0.23200500
N	-2.51486230	1.97101180	0.04700500
C	4.13913770	1.56801180	3.08700500
N	2.24613770	0.54501180	1.64900500
O	1.65613770	1.34401180	3.92200500
S	2.44313770	1.70201180	2.75600500
C	6.46613770	1.87601180	3.37300500
N	6.07413770	0.60601180	3.69300500
O	2.13113770	3.00501180	2.18400500
S	5.26813770	2.84501180	2.88600500
C	8.32013770	3.40901180	3.20600500
N	4.64813770	0.40001180	3.51300500
O	7.75613770	4.52301180	3.17300500
C	9.69213770	3.19401180	2.61000500
N	7.74413770	2.33901180	3.76700500
Zn	1.53613770	1.05301180	-0.07899500
H	-5.83492320	-1.72187660	-4.03539170
H	-3.75315490	-2.90138020	1.25411060
H	-4.01855680	-1.46678090	0.25504020
H	-2.65138350	-3.91149850	-1.01449200
H	-2.39953100	-2.18586190	-1.39904760
H	-4.86600990	-4.36777750	-0.51144860
H	-8.37843550	-2.66674200	1.78445940
H	2.58919660	1.29884670	-2.94470230
H	5.18727930	4.31913460	-1.52466460
H	3.80592870	3.49116740	0.47416960
H	-0.36627290	0.44985480	-2.58548870
H	1.01795550	-3.52526980	-2.29508520
H	2.26116450	-2.19651440	-0.54892210
H	-2.85768230	4.00421590	-0.59956190
H	-1.43354120	0.14150140	0.52479960
H	-3.47547810	1.67987330	0.24104560
H	10.35663680	2.58989690	3.26298110
H	10.16366110	4.17083660	2.40697130
H	9.58103150	2.63558040	1.65579150
H	-5.08465420	-1.78722560	-1.93222540
H	-6.43456560	-1.22260930	0.04074130
H	-8.89025860	-1.52188140	0.49457800
H	-5.81646000	-3.34901900	-4.82025450
H	-7.32134640	-2.73334070	-4.10501000
H	-0.55489090	-2.11336760	-3.71112020
H	-0.07521320	3.98803760	-0.77317290
H	4.58564620	3.11494290	-3.68715980
H	-8.05630590	-0.89970910	1.97091430
H	3.11819300	-0.01943100	1.58423460
H	8.34794570	1.53193450	3.97689910

6FE0

C	3.90070690	-2.16803110	-2.80039940
C	2.80370690	-1.55303110	-2.28839940

N	4.54070690	-2.76703110	-1.74039940
C	3.84770690	-2.54403110	-0.63139940
N	2.79370690	-1.81103110	-0.93839940
C	-0.84529310	1.53596890	-2.37039940
C	0.22670690	1.24196890	-1.59939940
N	-1.63829310	0.41496890	-2.36539940
C	-1.08429310	-0.51403110	-1.60839940
N	0.05070690	-0.04003110	-1.13639940
C	-2.72929310	7.38796890	-1.53439940
C	-3.55329310	6.55196890	-0.56139940
O	-4.78429310	6.56696890	-0.61839940
N	-2.87729310	5.75396890	0.26660060
C	-3.56029310	4.90896890	1.25160060
C	-3.77629310	5.71196890	2.53360060
O	-4.88529310	5.76896890	3.05960060
C	-2.75829310	3.62896890	1.55060060
C	-2.71129310	2.65496890	0.38660060
C	-2.80129310	1.18696890	0.79760060
O	-3.81529310	0.54596890	0.47260060
O	-1.84729310	0.66096890	1.41460060
N	-2.69629310	6.30096890	3.03460060
C	-2.76929310	7.19396890	4.17260060
C	3.32970690	1.36696890	1.01160060
C	3.08970690	2.59996890	1.51760060
N	2.10870690	0.72596890	0.94660060
C	1.17570690	1.55496890	1.40060060
N	1.74270690	2.69996890	1.72960060
Zn	1.32570690	-0.93603110	0.22160060
C	-0.26729310	-4.74503110	-1.80639940
C	-0.07129310	-4.09303110	-0.49739940
C	1.10970690	-4.38403110	0.34760060
C	2.15570690	-5.35703110	-0.17039940
C	1.92370690	-6.03603110	-1.48239940
C	0.72970690	-5.71403110	-2.30439940
N	3.13970690	-5.70503110	0.68260060
S	1.22170690	-3.59503110	1.76760060
O	2.57370690	-3.38803110	2.19660060
O	0.45370690	-4.36303110	2.72260060
N	0.64670690	-2.08703110	1.60760060
S	0.47870690	-6.45903110	-3.81439940
C	-0.34329310	-8.00703110	-3.63239940
C	3.54370690	-7.08903110	0.96060060
H	-1.62834140	7.29518260	-1.42469230
H	-3.25877530	3.15043770	2.41880870
H	-1.74037870	3.89671340	1.92111230
H	-1.78219660	2.80662070	-0.20545480
H	-3.56196450	2.85797800	-0.29415610
H	-4.56821050	4.67343230	0.86224990
H	-3.72139750	6.98895140	4.69641880
H	2.02109980	-0.96314180	-2.77431530
H	5.39303800	-3.32869420	-1.80660330
H	4.08677370	-2.92795820	0.36676290
H	1.10797000	1.83939980	-1.35212830
H	-2.58745930	0.36428310	-2.74691030
H	-1.53273280	-1.48467800	-1.37997200
H	3.77337470	3.42724020	1.73489240
H	0.06381880	1.34007190	1.45266270
H	1.25443050	3.50256870	2.13415170
H	0.88331440	-1.63528020	2.51724120
H	-1.35593270	-7.91116570	-3.19118300
H	-0.44974260	-8.37280710	-4.67263480

H	3.88627300	-7.60350050	0.04437690
H	-3.01866020	7.10513940	-2.56516520
H	-3.00831430	8.45216180	-1.40574480
H	-2.76794920	8.26324630	3.86704250
H	-1.92374220	7.02227690	4.86957890
H	4.26544280	0.89508240	0.69896550
H	-1.12791850	2.43928790	-2.91845420
H	4.29256180	-2.22995280	-3.82045840
F	2.80956220	-6.92782020	-1.91382600
F	-1.34824170	-4.44350080	-2.51406810
F	-1.02881490	-3.24138460	-0.06372160
H	3.20928660	-5.09702420	1.50620750
H	4.38198310	-7.07201520	1.68072930
H	2.70499070	-7.67194590	1.40117660
H	0.24543360	-8.75176380	-3.05937670
H	-1.86362610	5.67430810	0.17102350
H	-1.87406380	6.37448380	2.43399880

2O6E

C	-2.2221806	2.9475299	-2.1896431
C	-1.0651806	2.5685299	-1.5936431
N	-3.0641806	1.8605299	-2.1456431
C	-2.4491806	0.8675299	-1.5166431
N	-1.2451806	1.2755299	-1.1656431
C	-0.4661806	-3.0814701	-2.9686431
C	-0.6651806	-2.5454701	-1.7346431
N	0.6788194	-2.4944701	-3.4586431
C	1.1428194	-1.6234701	-2.5766431
N	0.3548194	-1.6474701	-1.5166431
C	-0.6341806	-3.0874701	1.4853569
C	-1.8151806	-2.1354701	1.5583569
O	-2.6271806	-2.3014701	2.4893569
O	-1.9441806	-1.2234701	0.6783569
C	2.2378194	2.4665299	0.7673569
S	2.2298194	1.4575299	-0.7026431
C	3.9738194	1.0095299	-0.7296431
C	0.1498194	0.3455299	4.1243569
C	0.5748194	-0.2144701	2.9663569
N	-0.7051806	1.3735299	3.7623569
C	-0.7931806	1.4225299	2.4443569
N	-0.0121806	0.4795299	1.9413569
Zn	-0.3331806	-0.2044701	-0.0276431
H	2.4981199	1.8791776	1.6715046
H	1.2163441	2.8691246	0.9072726
H	4.6033072	1.9181059	-0.8174980
H	4.2645964	0.4443700	0.1791627
H	4.1574866	0.3719724	-1.6161225
H	-0.1250724	3.1099162	-1.4507685
H	-4.0181998	1.8374950	-2.5134482
H	-2.8907538	-0.1011446	-1.2454298
H	-1.4726714	-2.6785232	-1.0108641
H	1.1029166	-2.7085459	-4.3646740
H	2.0271974	-0.9924032	-2.7110016
H	0.2651071	-2.6782612	0.9753618
H	-0.9285574	-3.9992808	0.9241102
H	1.2431812	-1.0624179	2.7942354
H	-1.2164600	1.9648062	4.4217687
H	-1.4258286	2.1067256	1.8701144
H	-1.0152913	-3.8371241	-3.5391107
H	-2.5160441	3.8840539	-2.6743154
H	0.3542915	0.1111222	5.1733199

H	-0.3655946	-3.4179400	2.5053648
H	2.9404291	3.3182303	0.6604408
